# Qualitative analysis of a discrete-time phytoplankton–zooplankton model with Holling type-II response and toxicity

**DOI:** 10.1186/s13662-021-03599-z

**Published:** 2021-10-09

**Authors:** Muhammad Salman Khan, Maria Samreen, Hassen Aydi, Manuel De la Sen

**Affiliations:** 1 Department of Mathematics, Quaid-I-Azam University, 45320 Islamabad, Pakistan; 2 Institut Supérieur d’Informatique et des Techniques de Communication, Université de Sousse, 4000 H. Sousse, Tunisia; 3 China Medical University Hospital, China Medical University, Taichung, 40402 Taiwan; 4 Department of Mathematics and Applied Mathematics, Sefako Makgatho Health Sciences University, Ga-Rankuwa, South Africa; 5 Faculty of Science and Technology, University of the Basque Country, 644 de Bilbao, Leioa, 48080 Bilbao Spain

**Keywords:** Phytoplankton–zooplankton model, Boundedness, Local stability analysis, Neimark–Sacker bifurcation, Generalized hybrid control method

## Abstract

The interaction among phytoplankton and zooplankton is one of the most important processes in ecology. Discrete-time mathematical models are commonly used for describing the dynamical properties of phytoplankton and zooplankton interaction with nonoverlapping generations. In such type of generations a new age group swaps the older group after regular intervals of time. Keeping in observation the dynamical reliability for continuous-time mathematical models, we convert a continuous-time phytoplankton–zooplankton model into its discrete-time counterpart by applying a dynamically consistent nonstandard difference scheme. Moreover, we discuss boundedness conditions for every solution and prove the existence of a unique positive equilibrium point. We discuss the local stability of obtained system about all its equilibrium points and show the existence of Neimark–Sacker bifurcation about unique positive equilibrium under some mathematical conditions. To control the Neimark–Sacker bifurcation, we apply a generalized hybrid control technique. For explanation of our theoretical results and to compare the dynamics of obtained discrete-time model with its continuous counterpart, we provide some motivating numerical examples. Moreover, from numerical study we can see that the obtained system and its continuous-time counterpart are stable for the same values of parameters, and they are unstable for the same parametric values. Hence the dynamical consistency of our obtained system can be seen from numerical study. Finally, we compare the modified hybrid method with old hybrid method at the end of the paper.

## Introduction

The study of mathematical models for population dynamics is considered as a key area in abstract ecology from the time when the famous Lotka–Volterra model was presented [1]. The learning of organism movement and spreading has turn out to be a fundamental element for understanding a chain of ecological interrogations associated with the spatiotemporal study of dynamics of populations [2]. Planktons are enormously flexible in abundance, both temporally and spatially. Plankton variability depends on natural along with physical procedure for the spatial structure. Natural processes include, for instance, development, grazing, and behavior, and physical procedures include, for instance, mixing and lateral stirring. Nonlinearity of ecosystems entirely contributes to the spatial organization in plankton allocations [3]. In marine ecology the word plankton refers to the spontaneously moving and faintly swimming organisms. Commonly, plankton is parted into two species, the phytoplankton species and zooplankton species. Phytoplankton species are tiny in their size with a single celled structure [4]. Phytoplankton are beneficial for aquatic life and produce half of the oxygen in the world through the process of photosynthesis. Phytoplankton population is exerting universal-scale effect on atmosphere by transporting $CO_{2}$ from water surface to the depth of oceans. Mainly, this process happens due to their death, sinking, and primary production [4]. It is observed that algal species rise abundantly in damped, wet, and marine environments. The stages of speedy growth, slow stagnation, and accelerated decline in the number of cells collectively create an algal bloom. This phenomenon of accelerated variation in the density of phytoplankton population is the central trait in the plankton ecosystem [4]. Despite the fact that the sudden emergence and disappearing of blooms is not clear, the undesirable effect of damaging algal blooms on the health of mankind, aquatic life, and fisheries trade can be easily seen [4].

On the incidence of blooms, phytoplankton and zooplankton interact with each other, and the study of this interaction is the point of focus of many scientific investigations [5]. Phytoplankton produces toxic materials to avert predations by their predators (zooplankton). Furthermore, this is the topic of interest of many researchers from many decades. Mathematical modeling of interactions between plankton species provides us an important optional method in improving the knowledge of any individual related to the biological and physical mechanisms concerning to the ecological study of plankton population [5].

The authors in [6] have considered a plankton–nutrient model related to aquatic environment by consideration of planktonic blooms. In [7] the authors have examined the influence of periodicity and seasonality on planktonic dynamics.

In [8] the authors have presented two mathematical models connected to plankton ecosystem along with a strong representation of viral septic phytoplankton and viruses. The authors in [9] have contemplated the effect of predation on competitory elimination and the coexistence of competitory predators. Moreover, they presented and explored a one-phytoplankton two-zooplankton model along with the consideration of harvesting.

Huppert et al. [10] considered a nutrient–phytoplankton model to examine the dynamical behavior of phytoplankton blooms. In [11] the authors have presented a zooplankton–phytoplankton model with harvesting. Furthermore, they have explained that the extra exploitation may exterminate the population while suitable harvesting guaranties the consolidation of both populations. Moreover, numerous studies have their point of focus on phytoplankton–zooplankton models along with a source of nutrient, the toxic consequence of plankton species, the survival of plankton species, or the harvesting effects [9–16]. It is convenient to introduce the toxin creating lag during the study of the dynamics of phytoplankton–zooplankton models. The authors in [17] have presented a mathematical model including time lag in toxin deliverance by phytoplankton. The work done in [18–21] motivated us to study the dynamics of a phytoplankton–zooplankton population model with toxicity. Moreover, the toxic substance is released by phytoplankton and sometimes by other external sources.

We consider the basic phytoplankton–zooplankton model presented by Chattopadhayay et al. [22]. Furthermore, this mathematical model is based on the following conditions.

- We suppose that $z(t)$ and $p(t)$ are the sizes of zooplankton and phytoplankton populations, respectively.
- Zooplankton population eats phytoplankton population and then recycles them into their own community. The functional response $\frac{\alpha p(t)z(t)}{a+p(t)}$ represents the predation rate of zooplankton population on phytoplankton species. Moreover, this predation increases the growth rate of zooplankton, which is represented by the term $\frac{\beta p(t)z(t)}{a+p(t)}$.
- We assume that zooplankton population becomes infected by eating infected phytoplankton population. Additionally, the infection in phytoplankton may be produced due to external toxic substance (see [22]).
- We assume that the infection in phytoplankton may be produced due to external toxic substance (see [22]).
- Phytoplankton population has logistic growth [21] in the absence of zooplankton population, where *r* is their exponential rate of growth, and *k* is the maximum carrying capacity of environment.

Under these conditions we have the following phytoplankton–zooplankton model [22]:

1.1 $$\begin{aligned} \textstyle\begin{cases} \frac{dp}{dt}=rp(t)(1-\frac{p(t)}{k})-\alpha f(p(t))z(t), \\ \frac{dz}{dt}=\beta f(p(t))z(t)-\delta z(t)-\rho g(p(t))z(t). \end{cases}\displaystyle \end{aligned}$$

Kuang [23] have inspected the limit cycle behavior in Gause-type predator–prey systems with Holling type-II response [24]. In addition, he revealed that the study of dynamical properties of predator–prey models using a Holling-type response function is better than the study of dynamics of predator–prey models without using Holling response. Generally, Holling type-II response is modeled and described by using rectangular hyperbola, and its mathematical form is given as

$$\begin{aligned} \varphi (x)=\frac{x}{a+x}, \end{aligned}$$

where *a* is any constant. By using Holling type-II response we get the following mathematical form of system (1.1):

1.2 $$\begin{aligned} \textstyle\begin{cases} \frac{dp}{dt}=rp(t)(1-\frac{p(t)}{k})-\alpha \frac{p(t)}{a+p(t)}z(t), \\ \frac{dz}{dt}=\beta \frac{p(t)}{a+p(t)} z(t)-\delta z(t)-\rho \frac{p(t)}{a+p(t)}z(t). \end{cases}\displaystyle \end{aligned}$$

- Next, we assume that the time lag for production and mediation of toxic substance by phytoplankton is zero.
- We introduce the catchability coefficients $q_{1}$ and $q_{2}$ for phytoplankton and zooplankton populations respectively. Generally, functional form for harvesting is expressed by using the hypothesis of catch-per-unit-effort [25].
- Moreover, we introduce *E* as the parameter for combined effort for harvesting of population [25].

Under these modifications, system (1.2) takes the following mathematical form:

1.3 $$\begin{aligned} \textstyle\begin{cases} \frac{dp}{dt}=rp(t)(1-\frac{p(t)}{k})-\alpha \frac{p(t)}{a+p(t)}z(t)-m_{1}p^{3}(t)-q_{1}Ep(t), \\ \frac{dz}{dt}=\beta \frac{p(t)}{a+p(t)} z(t)-\delta z(t)-\rho \frac{p(t)}{a+p(t)}z(t)-m_{2}z^{2}(t)-q_{2}Ez(t), \end{cases}\displaystyle \end{aligned}$$

where the parameters in system (1.3) are nonnegative and defined as follows:

*a*: constant of partial capturing saturation.

*α*: maximal takeover rate of zooplankton on phytoplankton.

*β*: conversion rate of phytoplankton–zooplankton $(\beta <\alpha )$.

*ρ*: toxicity rate of phytoplankton per unit biomass.

*δ*: natural rate of death of zooplankton population.

Moreover, the term $m_{1}p^{3}(t)$ appearing in system (1.3) represents the infection produced in phytoplankton population due to an external toxic substance. In addition, $\frac{d^{2}}{dp^{2}}(m_{1}p^{3})=6m_{1}p>0$ shows an accelerating growth of toxic substance parallel to phytoplankton population. This is due to fact that approximately each individual in phytoplankton population is increasingly consuming the toxic substances. However, the reduction of grazing by zooplankton due toxicity effect is represented by the term $m_{2}z^{2}(t)$. Furthermore, the toxicity effect on zooplankton population is less than phytoplankton population, where $m_{1}$ and $m_{2}$ are the toxicity coefficients with $0< m_{2}< m_{1}$ [25].

Obviously, it is appropriate to explore the dynamics of any biological model by difference equations instead of differential equations when we are dealing with nonoverlapping populations. Furthermore, observation and analysis of chaos in any biological system by using difference equations is better than by using differential equations [26]. Hence it is interesting to study biological models in discrete form. Recently, Ghanbari and Gómez-Aguilar [27] discussed the dynamics of nutrient–phytoplankton–zooplankton system with variable-order fractional derivatives. Moreover, the authors in [28] explored the existence of chaos in a cancer model using fractional derivatives by means of exponential decay and the Mittag-Leffler law. Beigi et al. [29] discussed the use of reinforcement learning for effective vaccination strategies of coronavirus disease 2019 (COVID-19). The authors in [30] analyzed the role of zooplankton dynamics for Southern Ocean phytoplankton biomass and global biogeochemical cycles. For more detail on the analysis of various dynamical systems, we refer the interested reader to [31–37]. There are various mathematical techniques for converting the systems of differential equations to their corresponding discrete counterparts. To achieve this goal, the usual way is applying standard difference schemes such as Runge–Kutta methods and Euler approximations. However, numerical inconsistency is experienced with the application of usual finite difference methods. Hence, to avoid this numerical inconsistency, we can apply the nonstandard finite difference method given by Mickens [38].

In general, whenever a nonstandard finite difference scheme is proposed, it is aimed on the preservation of the following properties of the respective continuous-time system: positivity of results, boundedness, stability of equilibrium points, and bifurcations. Moreover, the formation of these type of difference schemes is not straightforward, and there are no usual ways for their construction, which is probably considered as major downside of nonstandard difference schemes. Hence by taking into account the original dynamical properties of model (1.3) a discrete-time model from (1.3) is obtained by using Mickens-type nonstandard scheme such that it remains dynamically consistent [39]. Implementing the Mickens-type nonstandard scheme on model (1.3), we get the following discrete-time mathematical model:

1.4 $$\begin{aligned} \textstyle\begin{cases} \frac{p_{n+1}-p_{n}}{h}=rp_{n}(1-\frac{p_{n+1}}{k})-\alpha \frac{p_{n+1}}{a+p_{n}}z_{n}-m_{1}p_{n}^{2}p_{n+1}-q_{1}Ep_{n+1}, \\ \frac{z_{n+1}-z_{n}}{h}=\beta \frac{p_{n}}{a+p_{n}} z_{n}-\delta z_{n+1}- \rho \frac{p_{n}}{a+p_{n}}z_{n+1}-m_{2}z_{n}z_{n+1}-q_{2}Ez_{n+1}, \end{cases}\displaystyle \end{aligned}$$

where $h>0$ is taken as a step size for the nonstandard scheme. Furthermore, (1.4) can be written into the following mathematical form:

1.5 $$\begin{aligned} \textstyle\begin{cases} p_{n+1}= \frac{(1+hr)p_{n}}{1+h(\frac{r}{k}p_{n}+\frac{\alpha z_{n}}{a+p_{n}}+m_{1}p^{2}_{n}+q_{1}E)}, \\ z_{n+1}= \frac{(1+h\frac{\beta p_{n}}{a+p_{n}})z_{n}}{1+h(\frac{\rho p_{n}}{a+p_{n}}+\delta +m_{2}z_{n}+q_{2}E)}, \end{cases}\displaystyle \end{aligned}$$

where $\beta >\rho $. Moreover, our model (1.5) loses its biological consistency whenever $\beta <\rho $ (see [25]), which is impossible biologically. Hence, for the rest of our paper, we assume that $a>k$ and $\beta >\rho $.

## Boundedness and existence of fixed points for system (1.5)

To obtain steady states of system (1.5), we consider the following two-dimensional system of equations:

2.1 $$\begin{aligned} \textstyle\begin{cases} \underline{p}= \frac{(1+hr)\underline{p}}{1+h(\frac{r}{k}\underline{p}+\frac{\alpha \underline{z}}{a+\underline{p}}+m_{1}\underline{p}^{2}+q_{1}E)}, \\ \underline{z}= \frac{(1+h\frac{\beta \underline{p}}{a+\underline{p}})\underline{z}}{1+h (\frac{\rho \underline{p}}{a+\underline{p}}+\delta +m_{2}\underline{z}+q_{2}E)}. \end{cases}\displaystyle \end{aligned}$$

Solving (2.1), we can get the following equilibrium points: $(0,0)$ which is an extinction point for both populations, $(\frac{\sqrt{4 k^{2} m r+r^{2}-4 E k^{2} m q_{1}}-r}{2 k m}, 0)$, which is an extinction equilibrium for zooplankton population, and the unique positive equilibrium $(\underline{p},\underline{z})$. Additionally, the first component of the point $(\frac{\sqrt{4 k^{2} m r+r^{2}-4 E k^{2} m q_{1}}-r}{2 k m}, 0)$ remains positive for $r>Eq_{1}$. The existence and uniqueness of $(\underline{p},\underline{z})$ can be studied as follows. Suppose that $p_{0}>0$ and $z_{0}>0$. Then each solution ${(p_{n},z_{n})}$ of system (1.5) must satisfy $p_{n}>0$ and $z_{n}>0$ for all $n\geq 0$. Then from the first equation of system (1.5) it follows that

2.2 $$\begin{aligned} p_{n+1}\leq \frac{(1+hr)p_{n}}{1+\frac{hr}{k}p_{n}}. \end{aligned}$$

Consequently, solving (2.2) and then taking the limit, we get

2.3 $$\begin{aligned} \limsup_{n \to \infty }{p_{n}}\leq k. \end{aligned}$$

In the same way, from second equation of system (1.5) we get

$$\begin{aligned} z_{n+1}&= \frac{(1+h\frac{\beta p_{n}}{a+p_{n}})z_{n}}{1+h(\frac{\rho p_{n}}{a+p_{n}}+\delta +m_{2}z_{n}+q_{2}E)} \\ &\leq \frac{(1+h\frac{\beta k}{a+k})z_{n}}{1+h(\frac{\rho k}{a+k}+m_{2}z_{n})}. \end{aligned}$$

Hence, we can obtain the upper bound for zooplankton population:

2.4 $$\begin{aligned} \limsup_{n \to \infty }{z_{n}}\leq \frac{k(\beta -\rho )}{m_{2}(k+a)}. \end{aligned}$$

Finally, we have the following theorem about the boundedness of all solutions of (1.5).

### Theorem 2.1

*Assume that* $0< p_{0}\leq k$ *and* $0< z_{0}\leq \frac{k(\beta -\rho )}{m_{2}(k+a)}$. *Then for all* $n\geq 0$, *every positive solution* ${(p_{n},z_{n})}$ *of system* (1.5) *is bounded and contained in the set* $[0,k ]\times [0,\frac{k(\beta -\rho )}{m_{2}(k+a)} ]$ *whenever* $\beta >\rho $.

Next, we consider the equation system

2.5 $$\begin{aligned} \textstyle\begin{cases} \underline{p}= \frac{(1+hr)\underline{p}}{1+h(\frac{r}{k}\underline{p}+\frac{\alpha \underline{z}}{a+\underline{p}}+m_{1}\underline{p}^{2}+q_{1}E)}, \\ \underline{z}= \frac{(1+h\frac{\beta \underline{p}}{a+\underline{p}})\underline{z}}{1+h(\frac{\rho \underline{p}}{a+\underline{p}}+\delta +m_{2}\underline{z}+q_{2}E)}. \end{cases}\displaystyle \end{aligned}$$

From (2.5) we get the following pair:

$$\begin{aligned} \underline{p}= \frac{a(\beta -\rho )}{(\beta -\rho )-(\delta +m_{2}\underline{z}+q_{2}E)}-a, \qquad\underline{z}= \frac{(a+\underline{p})(r-\frac{r\underline{p}}{k}-m_{1}\underline{p}^{2}-q_{1}E)}{\alpha }. \end{aligned}$$

From this pair we can write

2.6 $$\begin{aligned} F(\underline{p})= \frac{a(\beta -\rho )}{(\beta -\rho )-(\delta +m_{2}f(\underline{p})+q_{2}E)}-a- \underline{p}, \end{aligned}$$

where

2.7 $$\begin{aligned} f(\underline{p})= \frac{(a+\underline{p})(r-\frac{r\underline{p}}{k}-m_{1}\underline{p}^{2}-q_{1}E)}{\alpha }, \end{aligned}$$

with

$$\begin{aligned} f(0)=\frac{a(r-q_{1}E)}{\alpha }>0 \end{aligned}$$

and

$$\begin{aligned} F(0)= \frac{a(\delta +m_{2}f(0)+q_{2}E)}{(\beta -\rho )-(\delta +m_{2}f(0)+q_{2}E)}>0. \end{aligned}$$

Furthermore, at the upper bound and for each $\lambda \in (0,k ]$, if $(\beta -\rho )>(\delta +m_{2}f(\lambda )+q_{2}E)$, then

$$\begin{aligned} F(\lambda )= \frac{a(\delta +m_{2}f(\lambda )+q_{2}E)}{(\beta -\rho )-(\delta +m_{2}f(\lambda )+q_{2}E)}- \lambda < 0, \end{aligned}$$

where

$$\begin{aligned} f(\lambda )=-\frac{(a+\lambda )(m_{1}r^{2}+q_{1}E)}{\alpha }< 0. \end{aligned}$$

Hence $F(\underline{p})=0$ has at least one positive real root in $[0,k ]$. Furthermore, we can see that

$$\begin{aligned} F'(\lambda )=-1+ \frac{a(\beta -\rho )(m_{2}f'(\lambda ))}{ ((\beta -\rho )-(\delta +m_{2}f(\lambda )+q_{2}E) )^{2}}< 0, \end{aligned}$$

where

$$\begin{aligned} f'(\lambda )=-\frac{(a+\lambda )(\frac{r}{k}+2m_{1}\lambda )}{\alpha }+ \frac{r-\frac{r\lambda }{k}-m_{1}\lambda ^{2}-q_{1}E}{\alpha }< 0, \end{aligned}$$

whenever

$$\begin{aligned} r-q_{1}E< \biggl(\frac{r}{k}+m_{1}\lambda \biggr) \lambda \end{aligned}$$

for every $\lambda \in [0,k ]$. Hence the equation $F(\underline{p})=0$ has a unique positive solution in $[0,k ]$.

### Theorem 2.2

*Assume that* $0< p_{0}\leq k$ *and* $0< z_{0}\leq \frac{k(\beta -\rho )}{m_{2}(k+a)}$. *Then for*

$$\begin{aligned} r-q_{1}E< \biggl(\frac{r}{k}+m_{1}\lambda \biggr) \lambda \end{aligned}$$

*and*

$$\begin{aligned} r>q_{1}E, \end{aligned}$$

*there exists a unique positive constant solution* $(\underline{p},\underline{z})$ *of system* (1.5) *in* $[0,k ]\times [0,\frac{k(\beta -\rho )}{m_{2}(k+a)} ]$ *if and only if for each* $\lambda \in (0,k ]$, *we have*

$$\begin{aligned} (\beta -\rho )>\bigl(\delta +m_{2}f(\lambda )+q_{2}E\bigr). \end{aligned}$$

*In addition*, *for* $\lambda =0$,

$$\begin{aligned} (\beta -\rho )< \bigl(\delta +m_{2}f(\lambda )+q_{2}E\bigr). \end{aligned}$$

## Stability analysis of system (1.5) about its fixed points

To discuss the stability of system (1.5) about all its equilibrium points, we compute the variational matrix $V_{(\underline{p},\underline{z})}$ of system (1.5) about each of its fixed point $(\underline{p},\underline{z})$. The matrix $V_{(\underline{p},\underline{z})}$ is given by

$$\begin{aligned} V_{(\underline{p},\underline{z})}= \begin{bmatrix} j_{11}&j_{12} \\ j_{21}& j_{22} \end{bmatrix} . \end{aligned}$$

The characteristic polynomial $\mathbb{M}(\xi )$ of $V_{(\underline{p},\underline{z})}$ is

3.1 $$\begin{aligned} \mathbb{M}(\xi )=\xi ^{2}-\operatorname{Tr}\xi +Dt, \end{aligned}$$

where

$$\begin{aligned} \operatorname{Tr}=(j_{11}+j_{22}) \end{aligned}$$

and

$$\begin{aligned} Dt=j_{11}j_{22}-j_{12}j_{21}. \end{aligned}$$

The next lemma describes the conditions parallel to the Jurry condition for the stability of fixed points; see [40].

### Lemma 3.1

([40])

*Let* $\mathbb{M}(\xi )=\xi ^{2}-\operatorname{Tr}\xi +Dt$ *and* $\mathbb{M}(1)>0$. *If* $\xi _{1}$, $\xi _{2}$ *are the roots of* $\mathbb{M}(\xi )=0$, *then*:

(*a*) $|\xi _{1}|<1$ *and* $|\xi _{2}|<1$ *if and only if* $\mathbb{M}(-1)>0$ *and* $Dt<1$;

(*b*) $|\xi _{1}|>1$ *and* $|\xi _{2}|>1$ *if and only if* $\mathbb{M}(-1)>0$ *and* $Dt>1$;

(*c*) $|\xi _{1}|<1$ *and* $|\xi _{2}|>1$ *or*($|\xi _{1}|>1$ *and* $|\xi _{2}<|1$) *if and only if* $\mathbb{M}(-1)<0$;

(*d*) $\xi _{1}$ *and* $\xi _{2}$ *represent complex conjugates with* $|\xi _{1}|=1=|\xi _{2}|$ *if and only if* ${\operatorname{Tr}}^{2}-4Dt<0$ *and* $Dt=1$.

*If* $\xi _{1}$ *and* $\xi _{2}$ *are characteristic values of* (3.1), *then the point* $(\underline{p},\underline{z})$ *is sink if* $|\xi _{1}|<1$ *and* $|\xi _{2}|<1$. *Furthermore*, *it is locally asymptotically stable*. *The point* $(\underline{p},\underline{z})$ *is known as a source* (*repeller*) *if* $|\xi _{1}|>1$ *and* $|\xi _{2}|>1$, *and it provides instability condition for the given system*. *The point* $(\underline{p},\underline{z})$ *is a saddle point if* $|\xi _{1}|<1$ *and* $|\xi _{2}|>1$ *or* $(|\xi _{1}|>1\textit{ and }|\xi _{2}|<1 )$. *Finally*, $(\underline{p},\underline{z})$ *is nonhyperbolic if condition* $(d)$ *is satisfied*.

Firstly, we will study the stability of system (1.5) about population free equilibrium point $(0,0)$. The variational matrix $V_{(\underline{p},\underline{z})}$ for system (1.5) evaluated at $(0,0)$ is

$$\begin{aligned} V_{(0,0)} = \begin{pmatrix} \frac{1+h r}{1+E h q_{1}} & 0 \\ 0 & \frac{1}{1+h \delta +E h q_{2}} \end{pmatrix}. \end{aligned}$$

Furthermore, $V_{(0,0)}$ is a diagonal matrix. Hence system (1.5) has two eigenvalues related to the population free equilibrium point $(0,0)$, $\xi _{1}= \frac{1+h r}{1+E h q_{1}}$ and $\xi _{2}=\frac{1}{1+h \delta +E h q_{2}}$, where, $\xi _{1}$ and $\xi _{2}$ are roots of the characteristic equation of the matrix $V_{(0,0)}$. It is clear that $|\xi _{2}|=|\frac{1}{1+h \delta +E h q_{2}}|<1$ for all parametric values. Now by considering the condition $|\xi _{2}|<1$ we are now able to describe stability conditions for system (1.5) about $(0,0)$.

### Proposition 3.2

*Let* $\xi _{1}$ *and* $\xi _{2}$ *be the roots of the characteristic equation of the matrix* $V_{(0,0)}$ *and suppose that* $|\xi _{2}|<1$ *for all parametric values*. *Let* $(0,0)$ *be a population free fixed point of system* (1.5). *Then* $(0,0)$ *is sink or saddle if and only if* $r< Eq_{1}$ *or* $r>Eq_{1}$, *respectively*.

Next, we will explore the local stability of system (1.5) about the zooplankton-free equilibrium $(\frac{\sqrt{4 k^{2} m r+r^{2}-4 E k^{2} m q_{1}}-r}{2 k m}, 0)$. Clearly, the first component in the pair $(\frac{\sqrt{4 k^{2} m r+r^{2}-4 E k^{2} m q_{1}}-r}{2 k m}, 0)$ is positive if and only if $r>q_{1} E$. Let $V_{1}(\frac{\sqrt{4 k^{2} m r+r^{2}-4 E k^{2} m q_{1}}-r}{2 k m}, 0)$ be the variational matrix of the two-dimensional system (1.5) about the zooplankton free equilibrium $(\frac{\sqrt{4 k^{2} m r+r^{2}-4 E k^{2} m q_{1}}-r}{2 k m}, 0)$. Then $V_{1}(\frac{\sqrt{4 k^{2} m r+r^{2}-4 E k^{2} m q_{1}}-r}{2 k m}, 0)$ has the following form:

$$\begin{aligned} V_{1}(\underline{x}, 0)= \begin{pmatrix} \frac{k^{2} (1+h r) (1+E h q_{1}-h m_{1} \underline{x}^{2} )}{ (k+E h k q_{1}+h \underline{x} (r+k m_{1} \underline{x} ) )^{2}} & - \frac{h k^{2} (1+h r) \alpha \underline{x}}{ (a+\underline{x} ) (k+E h k q_{1}+h \underline{x} (r+k m_{1} \underline{x} ) )^{2}} \\ 0 & \frac{a+\underline{x}+h \beta \underline{x}}{a+a h \delta +\underline{x}+h (\delta +\rho ) \underline{x}+E h q_{2} (a+\underline{x} )} \end{pmatrix}, \end{aligned}$$

where $\underline{x}= \frac{\sqrt{4 k^{2} m r+r^{2}-4 E k^{2} m q_{1}}-r}{2 k m}$. Moreover, $V_{1}(\underline{x}, 0)$ has the characteristic polynomial

3.2 $$\begin{aligned} \mathbb{M}(\xi )=\xi ^{2}-\operatorname{Tr} \bigl[V_{1}(\underline{x},0)\bigr]+\operatorname{Det}\bigl[V_{1}( \underline{x}, 0)\bigr] \end{aligned}$$

with

$$\begin{aligned} \begin{aligned} \operatorname{Tr}\bigl[V_{1}( \underline{x},0)\bigr]={}& \frac{a+\underline{x}+h \beta \underline{x}}{a+a h \delta +\underline{x}+h (\delta +\rho ) \underline{x}+E h q_{2} (a+\underline{x} )} \\ &{}+ \frac{k^{2} (1+h r) (1+E h q_{1}-h m_{1} \underline{x}^{2} )}{ (k+E h k q_{1}+h \underline{x} (r+k m_{1} \underline{x} ) )^{2}} \end{aligned} \end{aligned}$$

and

$$\begin{aligned} \begin{aligned} &\operatorname{Det}\bigl[V_{1}(\underline{x}, 0) \bigr]\\ &\quad= \frac{k^{2} (1+h r) (a+\underline{x}+h \beta \underline{x} ) (1+E h q_{1}-h m_{1} \underline{x}^{2} )}{ (a+a h \delta +\underline{x}+h (\delta +\rho ) \underline{x}+E h q_{2} (a+\underline{x} ) ) (k+E h k q_{1}+h \underline{x} (r+k m_{1} \underline{x} ) )^{2}}. \end{aligned} \end{aligned}$$

Hence we have the following proposition about the local stability of system (1.5) about the zooplankton-free equilibrium $( \frac{\sqrt{4 k^{2} m_{1} r+r^{2}-4 E k^{2} m_{1} q_{1}}-r}{2 k m_{1} }, 0)$.

### Proposition 3.3

*Let* $\xi _{1}$ *and* $\xi _{2}$ *be the characteristic roots of* *(3.2)*, *and let* $r>q_{1} E$. *If* $( \frac{\sqrt{4 k^{2} m_{1} r+r^{2}-4 E k^{2} m_{1} q_{1}}-r}{2 k m_{1} }, 0)=(\underline{x},0)$ *is a zooplankton*-*free constant solution of* (1.5), *then*:

(*a*) $(\underline{x},0)$ *remains inside the unit disk if and only if*

3.3 $$\begin{aligned} \bigl\vert 1+E h q_{1}-h m_{1} \underline{x}^{2} \bigr\vert < \frac{ (k+E h k q_{1}+h \underline{x} (r+k m_{1} \underline{x} ) )^{2}}{k^{2} (1+h r)} \end{aligned}$$

*and*

3.4 $$\begin{aligned} \beta \underline{x}< a \delta + (\delta +\rho ) \underline{x}+E q_{2} (a+\underline{x} ). \end{aligned}$$

(*b*) $(\underline{x},0)$ *lies outside the unit disk if and only if*

3.5 $$\begin{aligned} \bigl\vert 1+E h q_{1}-h m_{1} \underline{x}^{2} \bigr\vert > \frac{ (k+E h k q_{1}+h \underline{x} (r+k m_{1} \underline{x} ) )^{2}}{k^{2} (1+h r)} \end{aligned}$$

*and*

3.6 $$\begin{aligned} \beta \underline{x}>a \delta + (\delta +\rho ) \underline{x}+E q_{2} (a+\underline{x} ). \end{aligned}$$

(*c*) $(\underline{x},0)$ *is a saddle point if and only if one of the following pairs of inequalities* (3.4)*–*(3.5) *or* (3.3)*–*(3.6) *is satisfied*.

(*d*) $(\underline{x},0)$ *is nonhyperbolic if and only if one of the following conditions is satisfied*:

$$\begin{aligned} \bigl\vert 1+E h q_{1}-h m_{1} \underline{x}^{2} \bigr\vert = \frac{ (k+E h k q_{1}+h \underline{x} (r+k m_{1} \underline{x} ) )^{2}}{k^{2} (1+h r)} \end{aligned}$$

*or*

$$\begin{aligned} a \delta + (\delta +\rho -\beta ) \underline{x}+E q_{2} (a+ \underline{x} )=0. \end{aligned}$$

Finally, it remains to analyze the local stability of system (1.5) about the only positive fixed point $(\underline{p},\underline{z})$. Moreover, all parametric conditions for the existence of nonextinction fixed point $(\underline{p},\underline{z})$ are given in Theorems 2.1 and 2.2. We can calculate the Jacobian matrix $V_{2}(\underline{p},\underline{z})$ of system (1.5) about $(\underline{p},\underline{z})$ as follows:

$$\begin{aligned} V_{2}(\underline{p},\underline{z})= \begin{pmatrix} \frac{k^{2} (1+h r) ( (a+\underline{p} )^{2} (1+E h q_{1}-h m_{1} \underline{p}^{2} )+h \alpha (a+2 \underline{p} ) \underline{z} )}{ ( (a+\underline{p} ) (k+E h k q_{1}+h \underline{p} (r+k m_{1} \underline{p} ) )+h k \alpha \underline{z} )^{2}} & - \frac{h \alpha \underline{p}}{(1+h r) (a+\underline{p} )} \\ \frac{h \underline{z} (-\beta +\delta +\rho +E q_{2}+m_{2} \underline{z} )^{2}}{a (\beta +h \beta \delta -\rho +E h \beta q_{2}+h \beta m_{2} \underline{z} )} & \frac{\beta +h \beta \delta -\rho +E h \beta q_{2}+h \rho m_{2} \underline{z}}{\beta +h \beta \delta -\rho +E h \beta q_{2}+h \beta m_{2} \underline{z}} \end{pmatrix}, \end{aligned}$$

where

$$\begin{aligned} \underline{z}= \biggl(\frac{a+\underline{p}}{\alpha } \biggr) \biggl(r-r \frac{\underline{p}}{k}-m_{1} (\underline{p} )^{2}-q_{1}E \biggr) \end{aligned}$$

and

$$\begin{aligned} \underline{p}= \frac{a(\beta -\rho )}{(\beta -\rho )- (\delta +m_{2}\underline{z}+q_{2}E )}-a. \end{aligned}$$

Let $\mathbb{M}(\xi )$ be the characteristic polynomial for the matrix $V_{2}(\underline{p},\underline{z})$ with

$$\begin{aligned} &\sigma = (-\beta +\delta +\rho +Eq_{2} )\geq 0,\qquad \phi = (\beta +h \beta \delta -\rho +E h \beta ),\qquad (1+h r)= \eta q_{2}>0, \\ &\psi =\beta +\rho \quad\text{and}\quad \theta = \bigl( (a+\underline{p} ) \bigl(k+e h k q_{1}+h \underline{p} (r+k m_{1} \underline{p} ) \bigr)+h k \alpha \underline{z} \bigr)^{2}. \end{aligned}$$

Then we have

$$\begin{aligned} \operatorname{Tr}=1- \frac{h \underline{p} ((a-k) r+E k q_{1}+\underline{p} (2 r+k m_{1} (2 a+3 \underline{p} ) ) )}{k \eta (a+\underline{p} )}+ \frac{\phi +h \rho m_{2} \underline{z}}{\phi +h \beta m_{2} \underline{z}} \end{aligned}$$

and

$$\begin{aligned} Dt={}&\frac{1}{a k \eta (a+\underline{p} ) (\phi +h\beta m_{2} \underline{z} )} \bigl(a^{2} k \eta (\phi +h \rho m_{2} \underline{z} ) \bigr) \\ &{}-\frac{1}{a k \eta (a+\underline{p} ) (\phi +h\beta m_{2} \underline{z} )} \bigl(2 a h \underline{p}^{2} (r+a k m_{1} ) (\phi +h \rho m_{2} \underline{z} ) \bigr) \\ &{}+\frac{ak\underline{p}}{a k \eta (a+\underline{p} ) (\phi +h \beta m_{2} \underline{z} )} \biggl(\biggl(a \frac{h}{k} r-hr-\eta \biggr) \phi +E h q_{1} (\phi +h \rho m_{2} \underline{z} ) \biggr) \\ &{}-\frac{ak\underline{p}}{a k \eta (a+\underline{p} ) (\phi +h \beta m_{2} \underline{z} )} \bigl(3 h m_{1} \underline{p}^{2} ( \phi +h \rho m_{2} \underline{z} ) \bigr) \\ &{}+\frac{h \underline{z}\underline{p}}{a k \eta (a+\underline{p} ) (\phi +h \beta m_{2} \underline{z} )} \bigl( \bigl(a (-a h r+h k r+k \eta ) \rho +h k \alpha \underline{z} (2 \sigma +m_{2} \underline{z} ) \bigr) \bigr) \\ &{}+\frac{h \underline{z}\underline{p}}{a k \eta (a+\underline{p} ) (\phi +h \beta m_{2} \underline{z} )} \bigl(h k \alpha \sigma ^{2} \bigr). \end{aligned}$$

Taking into account the work done in the previous section, by Theorem 2.2 it follows that

$$\begin{aligned} \mathbb{M}(1)={}&\frac{h^{2} \underline{p} \underline{z} (m_{2} (a (\beta -\rho ) (E k q_{1}+\underline{p} (2 r+k m_{1} (2 a+3 \underline{p} ) ) )+2 k \alpha \sigma \underline{z}+k \alpha m_{2} \underline{z}^{2} ) )}{a k \eta (a+\underline{p} ) (\phi +h \beta m_{2} \underline{z} )} \\ &{}+\frac{h^{2} \underline{p} \underline{z} (k \alpha \sigma ^{2}+m_{2} a (\beta -\rho ) (a-k) r )}{a k \eta (a+\underline{p} ) (\phi +h \beta m_{2} \underline{z} )}>0 \end{aligned}$$

and

$$\begin{aligned} \mathbb{M}(-1)={}&\frac{-2a\phi \underline{p}}{a k \eta (a+\underline{p} ) (\phi +h \beta m_{2} \underline{z} )} \bigl(2 k \eta -E h k q_{1}-h \underline{p} \bigl(2 r+k m_{1} (2 a+3 \underline{p} ) \bigr) \bigr) \\ &{}+ \frac{2 a k \eta -\underline{p} (h (a-k) r )}{a k \eta (a+\underline{p} ) (\phi +h \beta m_{2} \underline{z} )} (2a\phi +h \underline{z} a \psi m_{2} ) \\ &{}+\frac{h \underline{z} a \psi m_{2} \underline{p}}{a k \eta (a+\underline{p} ) (\phi +h \beta m_{2} \underline{z} )} \bigl(2 k \eta -E h k q_{1}-h \underline{p} \bigl(2 r+k m_{1} (2 a+3 \underline{p} ) \bigr) \bigr) \\ &{}+\frac{h^{2} k \alpha \sigma \underline{p}\underline{z}}{a k \eta (a+\underline{p} ) (\phi +h \beta m_{2} \underline{z} )} \bigl(\sigma +2 m_{2} \underline{z}+ m_{2}^{2} \underline{z}^{2} \bigr). \end{aligned}$$

Hence the local stability of system (1.5) about $(\underline{p},\underline{z})$ can be studied with the help of the following proposition.

### Proposition 3.4

*Let* $a>k$ *and* $\beta >\rho $. *Then* $(\underline{p},\underline{z})$ *is a positive constant solution of* (1.5). *In addition*, *if*

$$\begin{aligned} \kappa =2 a k \eta -\underline{p} \bigl(h (a-k) r-2 k \eta +E h k q_{1}+h \underline{p} \bigl(2 r+k m_{1} (2 a+3 \underline{p} ) \bigr) \bigr), \end{aligned}$$

*then*

(*a*) *The point* $(\underline{p},\underline{z})$ *remains inside the unit disk if and only if*

$$\begin{aligned} \kappa > \frac{h^{2} k \alpha \sigma \underline{p}\underline{z} (\sigma +2 m_{2} \underline{z}+ m_{2}^{2} \underline{z}^{2} )}{ (2a\phi +h\underline{z}a\psi m_{2} )} \quad \textit{and} \quad Dt< 1. \end{aligned}$$

(*b*) *The point* $(\underline{p},\underline{z})$ *is repeller if and only if*

$$\begin{aligned} \kappa > \frac{h^{2} k \alpha \sigma \underline{p}\underline{z} (\sigma +2 m_{2} \underline{z}+ m_{2}^{2} \underline{z}^{2} )}{ (2a\phi +h\underline{z}a\psi m_{2} )} \quad\textit{and}\quad Dt>1. \end{aligned}$$

(*c*) *The point* $(\underline{p},\underline{z})$ *is a saddle point if and only if*

$$\begin{aligned} \kappa < \frac{h^{2} k \alpha \sigma \underline{p}\underline{z} (\sigma +2 m_{2} \underline{z}+ m_{2}^{2} \underline{z}^{2} )}{ (2a\phi +h\underline{z}a\psi m_{2} )}. \end{aligned}$$

(*d*) *The point* $(\underline{p},\underline{z})$ *is nonhyperbolic if and only if*

3.7 $$\begin{aligned} &h=\frac{a \phi \underline{p} ((a-k) r+E k q_{1}+\underline{p} (2 r+k m_{1} (2 a+3 \underline{p} ) ) )+a k \eta (\beta -\rho ) m_{2} (a+\underline{p} ) \underline{z}}{\underline{p} \underline{z} (k \alpha \sigma ^{2}-m_{2} (a \rho ((a-k) r+E k q_{1}+\underline{p} (2 r+k m_{1} (2 a+3 \underline{p} ) ) ) -2 k \alpha \sigma \underline{z}-k \alpha m_{2} \underline{z}^{2} ) )}, \end{aligned}$$

3.8 $$\begin{aligned} &\begin{aligned} &h \underline{p} \bigl(E k q_{1}+ \underline{p} \bigl(2 r+k m_{1} (2 a+3 \underline{p} ) \bigr) \bigr) ( \phi +h \rho m_{2} \underline{z} )+h \underline{p}(a-k) r \\ &\quad\neq 2 (\phi +h \rho m_{2} \underline{z} )k \eta (a+ \underline{p} ), \end{aligned} \end{aligned}$$

*and*

3.9 $$\begin{aligned} \begin{aligned} &h \underline{p} \bigl((a-k) r+E k q_{1}+ \underline{p} \bigl(2 r+k m_{1} (2 a+3 \underline{p} ) \bigr) \bigr) ( \phi +h \rho m_{2} \underline{z} ) \\ &\quad \neq (\phi +h \rho m_{2} \underline{z} )k \eta (a+ \underline{p} ). \end{aligned} \end{aligned}$$

## Bifurcation analysis of positive equilibrium of system (1.5)

This section is related to the bifurcation analysis of system (1.5) about $(\underline{p},\underline{z})$, where all conditions for the existence and positivity of $(\underline{p},\underline{z})$ are given in Theorems 2.1 and 2.2. Here we will discuss the Neimark–Scaker bifurcation experienced by system (1.5) about $(\underline{p},\underline{z})$ under some conditions. Bifurcation is the mathematical phenomenon produced in any system due to creation of very small change in stability of the system. Furthermore, it causes some surprising changes in the dynamical standards of any mathematical system. Mathematically, bifurcation arises whenever parameters are varied in a very small neighborhood of an equilibrium point. Moreover, for further study of bifurcation theory and understanding this surprising behavior of a discrete-time mathematical system, we refer to [41–46]. Here we use standard theory of bifurcation for the study of Neimark–Sacker bifurcation of system (1.5) at $(\underline{p},\underline{z})$. Let $\xi _{1}$ and $\xi _{2}$ be the roots of (3.1). Then both roots are complex with modulus one if $(\underline{p},\underline{z})$ is a nonhyperbolic fixed point under condition $(d)$ of Proposition 3.4. Hence system (1.5) experiences the Neimark–Sacker bifurcation when the parameters in system (1.5) vary in a small neighborhood of the set

$$\begin{aligned} \mho _{*}= \bigl\{ \alpha,\beta,a,k,r,\delta,\rho, m_{1}, m_{2}, q_{1}, q_{2}, E\in \Re ^{+}: h\in (0,1) \bigr\} , \end{aligned}$$

and (3.8) and (3.9) are satisfied. Let $(\alpha,\beta,a,k,r,\delta,\rho, m_{1}, m_{2}, q_{1}, q_{2}, E )\in {\mho _{*}}$ with

$$\begin{aligned} h= \frac{a \phi \underline{p} ((a-k) r+E k q_{1}+\underline{p} (2 r+k m_{1} (2 a+3 \underline{p} ) ) )+a k \eta (\beta -\rho ) m_{2} (a+\underline{p} ) \underline{z}}{\underline{p} \underline{z} (k \alpha \sigma ^{2}-m_{2} (a \rho ((a-k) r+E k q_{1}+\underline{p} (2 r+k m_{1} (2 a+3 \underline{p} ) ) )-2 k \alpha \sigma \underline{z}-k \alpha m_{2} \underline{z}^{2} ) )}. \end{aligned}$$

Then system (1.5) can be written as

4.1 $$\begin{aligned} \begin{pmatrix} p \\ z \end{pmatrix}\to \begin{pmatrix} \frac{(1+hr)p}{1+h(\frac{r}{k}p+\frac{\alpha z}{a+p}+m_{1}p^{2}+q_{1}E)} \\ \frac{(1+h\frac{\beta p}{a+p})z}{1+h(\frac{\rho p}{a+p}+\delta +m_{2}z+q_{2}E)} \end{pmatrix}. \end{aligned}$$

Assume that $(\alpha,\beta,a,k,r,\delta,\rho, m_{1}, m_{2}, q_{1}, q_{2}, E )\in {\mho _{*}}$. Taking *h̆* as a bifurcation parameter, we get the following form of system (4.1):

4.2 $$\begin{aligned} \begin{pmatrix} p \\ z \end{pmatrix}\to \begin{pmatrix} \frac{(1+(h+\breve{h})r)p}{1+(h+\breve{h})(\frac{r}{k}p+\frac{\alpha z}{a+p}+m_{1}p^{2}+q_{1}E)} \\ \frac{(1+(h+\breve{h})\frac{\beta p}{a+p})z}{1+(h+\breve{h})(\frac{\rho p}{a+p}+\delta +m_{2}z+q_{2}E)} \end{pmatrix}, \end{aligned}$$

where $|\breve{h}|\ll 1 $ is a very small perturbation parameter. Next, we assume that

$$\begin{aligned} P=p-\underline{p},\qquad Z=z-\underline{z}. \end{aligned}$$

Then the map (4.1) takes the form

4.3 $$\begin{aligned} \begin{pmatrix} P \\ Z \end{pmatrix}\to \begin{pmatrix} v_{11} & v_{12} \\ v_{21}& v_{22} \end{pmatrix} \begin{pmatrix} P \\ Z \end{pmatrix}+ \begin{pmatrix} \check{f}(P,Z) \\ \check{g}(P,Z) \end{pmatrix}, \end{aligned}$$

where

$$\begin{aligned} \check{f}(P,Z)={}&v_{13}P^{2}+v_{14}PZ+v_{15}Z^{2}+v_{16}P^{3}+v_{17}P^{2}Z+v_{18}PZ^{2} \\ &{}+ v_{19}Z^{3}+O \bigl(\bigl( \vert P \vert + \vert Z \vert \bigr)^{4}\bigr), \\ \check{g}(P,Z)={}&v_{23}P^{2}+v_{24}PZ+v_{25}Z^{2}+v_{26}P^{3}+v_{27}P^{2}Z+v_{28}PZ^{2} \\ &{}+v_{29}Z^{3}+O \bigl(\bigl( \vert P \vert + \vert Z \vert \bigr)^{4}\bigr). \end{aligned}$$

Moreover, the coefficients $v_{ij}$ for $i,j=1,2,\ldots,9$ are

$$\begin{aligned} v_{11}={}&\frac{k^{2} (1+h^{*} r) ((a+\underline{p})^{2}+h^{*} (a+2 \underline{p}) \underline{z} \alpha +h^{*} (a+\underline{p})^{2} (-\underline{p}^{2} m_{1}+E q_{1} ) )}{ ((a+\underline{p}) (k+h^{*} \underline{p} r)+h^{*} k \underline{z} \alpha +h^{*} k (a+\underline{p}) (\underline{p}^{2} m_{1}+E q_{1} ) )^{2}}, \\ v_{12}={}&\frac{h^{*} \underline{p} (1+h^{*} r) \alpha }{(a+\underline{p}) (1+x_{1} )^{2}}, \\ v_{13}={}&\frac{{h^{*}}^{2} \underline{p} (1+{h^{*}} r) (\frac{r}{k}-\frac{\underline{z} \alpha }{(a+\underline{p})^{2}}+2 \underline{p} m_{1} )^{2}}{ (1+x_{1} )^{3}}- \frac{h \underline{p} (1+{h^{*}} r) (\frac{2 z \alpha }{(a+\underline{p})^{3}}+2 m_{1} )}{2 (1+x_{1} )^{2}} \\ &{}-\frac{{h^{*}} (1+{h^{*}} r) (\frac{r}{k}-\frac{\underline{z} \alpha }{(a+\underline{p})^{2}}+2 \underline{p} m_{1} )}{ (1+x_{1} )^{2}}, \\ v_{14}={}&\frac{2 {h^{*}}^{2} \underline{p} (1+{h^{*}} r) \alpha (\frac{r}{k}-\frac{\underline{z} \alpha }{(a+\underline{p})^{2}}+2 \underline{p} m_{1} )}{(a+ \underline{p}) (1+x_{1} )^{3}}+ \frac{{h^{*}} \underline{p} (1+{h^{*}} r) \alpha }{(a+ \underline{p})^{2} (1+x_{1} )^{2}} \\ &{}-\frac{{h^{*}} (1+{h^{*}} r) \alpha }{(a+\underline{p}) (1+x_{1} )^{2}}, v_{15}= \frac{{h^{*}}^{2} \underline{p} (1+{h^{*}} r) \alpha ^{2}}{(a+\underline{p})^{2} (1+x_{1} )^{3}}, \\ v_{16}={}&{-} \frac{{h^{*}}^{3} \underline{p} (1+{h^{*}} r) (\frac{r}{k}-\frac{\underline{z} \alpha }{(a+\underline{p})^{2}}+2 \underline{p} m_{1} )^{3}}{ (1+x_{1} )^{4}}- \frac{{h^{*}} (1+{h^{*}} r) (\frac{2 \underline{z} \alpha }{(a+\underline{p})^{3}}+2 m_{1} )}{2 (1+x_{1} )^{2}} \\ &{}+\frac{{h^{*}}^{2} (1+{h^{*}} r) (\frac{r}{k}-\frac{\underline{z} \alpha }{(a+\underline{p})^{2}}+2 \underline{p} m_{1} )^{2}}{ (1+x_{1} )^{3}}+ \frac{{h^{*}} \underline{p} (1+{h^{*}} r) \underline{z} \alpha }{(a+\underline{p})^{4} (1+x_{1} )^{2}} \\ &{}+\frac{{h^{*}}^{2} \underline{p} (1+{h^{*}} r) (\frac{2 \underline{z} \alpha }{(a+\underline{p})^{3}}+2 m_{1} ) (\frac{r}{k}-\frac{\underline{z} \alpha }{(a+\underline{p})^{2}}+2 \underline{p} m_{1} )}{ (1+x_{1} )^{3}}, \\ v_{17}={}&{-} \frac{3 {h^{*}}^{3} \underline{p} \alpha (1+{h^{*}} r) (\frac{r}{k}-\frac{\underline{z} \alpha }{(a+\underline{p})^{2}}+2 \underline{p} m_{1} )^{2}}{(a+\underline{p}) (1+x_{1} )^{4}}- \frac{{h^{*}} \underline{p} (1+{h^{*}} r) \alpha }{(a+\underline{p})^{3} (1+x_{1} )^{2}} \\ &{}-\frac{2 {h^{*}}^{2} \underline{p} \alpha (1+{h^{*}} r) (\frac{r}{k}-\frac{\underline{z} \alpha }{(a+\underline{p})^{2}}+2 \underline{p} m_{1} )}{(a+\underline{p})^{2} (1+x_{1} )^{3}}+ \frac{{h^{*}} (1+{h^{*}} r) \alpha }{(a+\underline{p})^{2} (1+x_{1} )^{2}} \\ &{}+\frac{{h^{*}}^{2} \underline{p}\alpha (1+{h^{*}} r) (\frac{2 \underline{z} \alpha }{(a+\underline{p})^{3}}+2 m_{1} )}{(a+\underline{p}) (1+x_{1} )^{3}}+ \frac{2 {h^{*}}^{2} \alpha (1+{h^{*}} r) (\frac{r}{k}-\frac{\underline{z} \alpha }{(a+\underline{p})^{2}}+2 \underline{p} m_{1} )}{(a+\underline{p}) (1+x_{1} )^{3}}, \\ v_{18}={}&{-} \frac{3 {h^{*}}^{3} \underline{p} (1+h r) \alpha ^{2} (\frac{r}{k}-\frac{\underline{z} \alpha }{(a+\underline{p})^{2}}+2 \underline{p} m_{1} )}{(a+\underline{p})^{2} (1+x_{1} )^{4}}- \frac{2 {h^{*}}^{2} \underline{p} (1+{h^{*}} r) \alpha ^{2}}{(a+\underline{p})^{3} (1+x_{1} )^{3}} \\ &{}+\frac{{h^{*}}^{2} (1+{h^{*}} r) \alpha ^{2}}{(a+\underline{p})^{2} (1+x_{1} )^{3}}, v_{19}=- \frac{{h^{*}}^{3} \underline{p} (1+{h^{*}} r) \alpha ^{3}}{(a+\underline{p})^{3} (1+x_{1} )^{4}}, \\ v_{21}={}&\frac{a {h^{*}} \underline{z} (\beta +{h^{*}} \beta \delta -\rho +{h^{*}} \underline{z} \beta m_{2}+E {h^{*}} \beta q_{2} )}{ (a+ \underline{p}+a {h^{*}} \delta +{h^{*}} \underline{p} (\delta +\rho )+{h^{*}} (a+ \underline{p}) ( \underline{z} m_{2}+E q_{2} ) )^{2}} , \\ v_{22}={}&\frac{(a+ \underline{p}+{h^{*}} \underline{p} \beta ) (a+ \underline{p}+a {h^{*}} \delta +{h^{*}} \underline{p} (\delta +\rho )+E {h^{*}} (a+\underline{p}) q_{2} )}{ (a+\underline{p}+a {h^{*}} \delta +{h^{*}} \underline{p} (\delta +\rho )+{h^{*}} (a+\underline{p}) (\underline{z} m_{2}+E q_{2} ) )^{2}}, \\ v_{23}={}&{-} \frac{a {h^{*}} \underline{z} (1+{h^{*}} (\delta +\rho )+{h^{*}} \underline{z} m_{2}+E {h^{*}} q_{2} ) (\beta +{h^{*}} \beta \delta -\rho +{h^{*}} \underline{z} \beta m_{2}+E {h^{*}} )}{ (a+\underline{p}+a {h^{*}} \delta +{h^{*}} \underline{p} (\delta +\rho )+{h^{*}} (a+\underline{p}) (\underline{z} m_{2}+E q_{2} ) )^{3}}, \\ v_{24}={}&\frac{2 {h^{*}}^{2} \underline{z} (1+\frac{{h^{*}} \underline{p} \beta }{a+\underline{p}} ) (-\frac{\underline{p} \rho }{(a+p)^{2}}+\frac{\rho }{a+\underline{p}} ) m_{2}}{ (1+x_{2} )^{3}}- \frac{{h^{*}} (1+\frac{{h^{*}} \underline{p} \beta }{a+\underline{p}} ) (-\frac{\underline{p} \rho }{(a+\underline{p})^{2}}+\frac{\rho }{a+\underline{p}} )}{ (1+x_{2} )^{2}} \\ &{}-\frac{{h^{*}} \underline{z} (-\frac{{h^{*}} \underline{p} \beta }{(a+\underline{p})^{2}}+\frac{{h^{*}} \beta }{a+\underline{p}} ) m_{2}}{ (1+x_{2} )^{2}}+ \frac{-\frac{{h^{*}} \underline{p} \beta }{(a+\underline{p})^{2}}+\frac{{h^{*}} \beta }{a+\underline{p}}}{1+x_{2}}, \\ v_{25}={}&\frac{{h^{*}} (a+\underline{p}) (a+\underline{p}+{h^{*}} \underline{p} \beta ) m_{2} (a+\underline{p}+a {h^{*}} \delta +{h^{*}} \underline{p} (\delta +\rho )+E {h^{*}} (a+\underline{p}) q_{2} )}{ (a+\underline{p}+a {h^{*}} \delta +{h^{*}} \underline{p} (\delta +\rho )+{h^{*}} (a+\underline{p}) (\underline{z} m_{2}+E q_{2} ) )^{3}}, \\ v_{26}={}&{-} \frac{{h^{*}}^{3} \underline{z} (1+\frac{{h^{*}} \underline{p} \beta }{a+\underline{p}} ) (-\frac{\underline{p} \rho }{(a+\underline{p})^{2}}+\frac{\rho }{a+\underline{p}} )^{3}}{ (1+x_{2} )^{4}}- \frac{{h^{*}} \underline{z} (1+\frac{{h^{*}} \underline{p} \beta }{a+\underline{p}} ) (\frac{6 \rho }{(a+\underline{p})^{3}} )}{6 (1+x_{2} )^{2}} \\ &{}+\frac{{h^{*}}^{2} \underline{z} (-\frac{{h^{*}} \underline{p} \beta }{(a+\underline{p})^{2}}+\frac{{h^{*}} \beta }{a+\underline{p}} ) (-\frac{\underline{p} \rho }{(a+\underline{p})^{2}}+\frac{\rho }{a+\underline{p}} )^{2}}{ (1+x_{2} )^{3}} \\ &{}-\frac{{h^{*}} \underline{z} (-\frac{{h^{*}} \underline{p} \beta }{(a+\underline{p})^{2}}+\frac{{h^{*}} \beta }{a+\underline{p}} ) (\frac{2 \underline{p} \rho }{(a+\underline{p})^{3}}-\frac{2 \rho }{(a+\underline{p})^{2}} )}{2 (1+x_{2} )^{2}}+ \frac{\underline{z} (-\frac{6 {h^{*}} \underline{p} \beta }{(a+\underline{p})^{4}}+\frac{6 {h^{*}} \beta }{(a+\underline{p})^{3}} )}{6 (1+x_{2} )} \\ &{}+\frac{{h^{*}}^{2} \underline{z} (1+\frac{{h^{*}} \underline{p} \beta }{a+\underline{p}} ) (\frac{2 \underline{p} \rho }{(a+\underline{p})^{3}}-\frac{2 \rho }{(a+\underline{p})^{2}} ) (-\frac{\underline{p} \rho }{(a+\underline{p})^{2}}+\frac{\rho }{a+\underline{p}} )}{ (1+x_{2} )^{3}} , \\ v_{27}={}&\frac{a {h^{*}} \underline{z} (1+{h^{*}} (\delta +\rho )+{h^{*}} \underline{z} m_{2}+E {h^{*}} q_{2} )^{2} ({h^{*}} \beta \delta -\rho +{h^{*}} \underline{z} \beta m_{2}+E {h^{*}} )}{ (a+\underline{p}+a {h^{*}} \delta +{h^{*}} \underline{p} (\delta +\rho )+{h^{*}} (a+\underline{p}) (\underline{z} m_{2}+E q_{2} ) )^{4}}, \\ v_{28}={}&{-} \frac{3 {h^{*}}^{3} \underline{z} (1+\frac{{h^{*}} \underline{p} \beta }{a+\underline{p}} ) (-\frac{\underline{p} \rho }{(a+\underline{p})^{2}}+\frac{\rho }{a+\underline{p}} ) m_{2}^{2}}{ (1+x_{2} )^{4}}- \frac{{h^{*}} (-\frac{{h^{*}} \underline{p} \beta }{(a+\underline{p})^{2}}+\frac{{h^{*}} \beta }{a+\underline{p}} ) m_{2}}{ (1+x_{2} )^{2}} \\ &{}+\frac{2 {h^{*}}^{2} (1+\frac{{h^{*}} \underline{p} \beta }{a+\underline{p}} ) (-\frac{\underline{p} \rho }{(a+\underline{p})^{2}}+\frac{\rho }{a+\underline{p}} ) m_{2}}{ (1+x_{2} )^{3}}+ \frac{{h^{*}}^{2} \underline{z} (-\frac{{h^{*}} \underline{p} \beta }{(a+\underline{p})^{2}}+\frac{{h^{*}} \beta }{a+\underline{p}} ) m_{2}^{2}}{ (1+x_{2} )^{3}}, \\ v_{29}={}&\frac{{h^{*}}^{2} (a+\underline{p})^{2} (a+\underline{p}+{h^{*}} \underline{p} \beta ) m_{2}^{2} (a {h^{*}} \delta +{h^{*}} \underline{p} (\delta +\rho )+E {h^{*}} (a+\underline{p}) q_{2} )}{ (a+\underline{p}+a {h^{*}} \delta +{h^{*}} \underline{p} (\delta +\rho )+{h^{*}} (a+\underline{p}) (\underline{z} m_{2}+E q_{2} ) )^{4}}, \end{aligned}$$

with

$$\begin{aligned} x_{1}={h^{*}} \biggl(\frac{\underline{p} r}{k}+ \frac{\underline{z} \alpha }{a+\underline{p}}+\underline{p}^{2} m_{1}+E q_{1} \biggr),\qquad x_{2}={h^{*}} \biggl(\delta + \frac{\underline{p} \rho }{a+\underline{p}}+\underline{z} m_{2}+E q_{2} \biggr), \end{aligned}$$

and

$$\begin{aligned} {h^{*}}=(h+\breve{h}). \end{aligned}$$

The characteristic equation $\mathbb{M}(\xi )=0$ obtained from Jacobian matrix of system (4.3) about $(0,0)$ is

4.4 $$\begin{aligned} \xi ^{2}-\operatorname{Tr}(\breve{h})\xi +Dt( \breve{h})=0, \end{aligned}$$

with

$$\begin{aligned} \operatorname{Tr}(\breve{h})=1- \frac{\bar{h} \underline{p} ((a-k) r+E k q_{1}+\underline{p} (2 r+k m_{1} (2 a+3 \underline{p} ) ) )}{k \eta (a+\underline{p} )}+ \frac{\phi +\bar{h}\rho m_{2} \underline{z}}{\phi +\bar{h} \beta m_{2} \underline{z}} \end{aligned}$$

and

$$\begin{aligned} Dt(\breve{h})={}&\frac{1}{a k \eta (a+\underline{p} ) (\phi +\bar{h}\beta m_{2} \underline{z} )} \bigl(a^{2} k \eta (\phi +\bar{h} \rho m_{2} \underline{z} ) \bigr) \\ &{}-\frac{1}{a k \eta (a+\underline{p} ) (\phi +\bar{h} \beta m_{2} \underline{z} )} \bigl(2 a \bar{h} \underline{p}^{2} (r+a k m_{1} ) ( \phi +\bar{h} \rho m_{2} \underline{z} ) \bigr) \\ &{}+\frac{ak\underline{p}}{a k \eta (a+\underline{p} ) (\phi +\bar{h} \beta m_{2} \underline{z} )} \biggl(\biggl(a \frac{\bar{h} }{k} r-\bar{h} r-\eta \biggr) \phi +E \bar{h} q_{1} (\phi +\bar{h} \rho m_{2} \underline{z} ) \biggr) \\ &{}-\frac{ak\underline{p}}{a k \eta (a+\underline{p} ) (\phi +\bar{h} \beta m_{2} \underline{z} )} \bigl(3 \bar{h} m_{1} \underline{p}^{2} (\phi +\bar{h} \rho m_{2} \underline{z} ) \bigr) \\ &{}+\frac{\bar{h} \underline{z}\underline{p}}{a k \eta (a+\underline{p} ) (\phi +\bar{h} \beta m_{2} \underline{z} )} \bigl( \bigl(a (-a \bar{h} r+\bar{h} k r+k \eta ) \rho + \bar{h} k \alpha \underline{z} (2 \sigma +m_{2} \underline{z} ) \bigr) \bigr) \\ &{}+\frac{\bar{h} \underline{z}\underline{p}}{a k \eta (a+\underline{p} ) (\phi +\bar{h} \beta m_{2} \underline{z} )} \bigl(\bar{h} k \alpha \sigma ^{2} \bigr), \end{aligned}$$

where $\bar{h}=h+\breve{h}$. As $(\alpha,\beta,a,k,r,\delta,\rho, m_{1}, m_{2}, q_{1}, q_{2}, E )\in {\mho _{*}}$, the roots of (4.4) are complex numbers $\xi _{1}$ and $\xi _{2}$ such that $|\xi _{1}|=|\xi _{2}|=1$. Then we can immediately see that

$$\begin{aligned} \check{\rho }(\breve{h})=\frac{\operatorname{Tr}(\breve{h})}{2}+\frac{i}{2}\sqrt{4Dt( \breve{h})-{\operatorname{Tr}}^{2}(\breve{h})}. \end{aligned}$$

Furthermore, we can have ${\check{\rho }}^{m}(0) \neq 1 $ for all $m\in \{1, 2, 3, 4\}$ if and only if

4.5 $$\begin{aligned} \operatorname{Tr}(\breve{h})=1- \frac{\bar{h} \underline{p} ((a-k) r+E k q_{1}+\underline{p} (2 r+k m_{1} (2 a+3 \underline{p} ) ) )}{k \eta (a+\underline{p} )}+ \frac{\phi +\bar{h}\rho m_{2} \underline{z}}{\phi +\bar{h} \beta m_{2} \underline{z}} \ne \pm 2,0,1. \end{aligned}$$

Since $(\alpha,\beta,a,k,r,\delta,\rho, m_{1}, m_{2}, q_{1}, q_{2}, E )\in {\mho _{*}}$ and (3.8)–(3.9) are satisfied, condition (4.5) is automatically satisfied, and

$$\begin{aligned} \vert \xi _{1} \vert = \bigl\vert \check{\rho }(\breve{h}) \bigr\vert =\sqrt{Dt(\breve{h})}, \biggl(\frac{d\sqrt{Dt(\breve{h})}}{d\breve{h}} \biggr)_{\breve{h}=0} \neq 0. \end{aligned}$$

Finally, to write the linear part of (4.3) in the canonical matrix form at $\breve{h}=0$, we consider the similarity transformation

4.6 $$\begin{aligned} \begin{pmatrix} P \\ Z \end{pmatrix}= \begin{pmatrix} v_{12} & 0 \\ \ell -v_{11} & -\wp \end{pmatrix} \begin{pmatrix} X \\ Y, \end{pmatrix}, \end{aligned}$$

where

$$\begin{aligned} \ell =\frac{\operatorname{Tr}(0)}{2}, \end{aligned}$$

and

$$\begin{aligned} \wp =\frac{\sqrt{4Dt(0)-\operatorname{Tr}^{2}(0)}}{2}. \end{aligned}$$

Then from (4.6) we have

4.7 $$\begin{aligned} \begin{pmatrix} X \\ Y \end{pmatrix}= \begin{pmatrix} \frac{1}{v_{12}} & 0 \\ \frac{\ell -v_{11}}{\wp v_{12} } & -\frac{1}{\wp } \end{pmatrix} \begin{pmatrix} P \\ Z. \end{pmatrix}. \end{aligned}$$

By using transformation (4.6) we have the next authoritative form of system (4.3):

4.8 $$\begin{aligned} \begin{pmatrix} X \\ Y \end{pmatrix}\to \begin{pmatrix} \ell & -\wp \\ \wp &\ell \end{pmatrix} \begin{pmatrix} X \\ Y \end{pmatrix}+ \begin{pmatrix} \breve{F}(X,Y) \\ \breve{G}(X,Y) \end{pmatrix}, \end{aligned}$$

where

$$\begin{aligned} \breve{F}(X,Y)={}&{\frac{v_{{16}}{P}^{3}}{v_{{12}}}}+{ \frac{v_{{17}}{P}^{2}Z}{v_{{12}} }}+{ \frac{v_{{13}}{P}^{2}}{v_{{12}}}}+{ \frac{v_{{18}}P{Z}^{2}}{v_{{ 12}}}}+{\frac{v_{{14}}PZ}{v_{{12}}}}+{ \frac{{Z}^{3}v_{{19}}}{v_{{12} }}}+{\frac{{Z}^{2}v_{{15}}}{v_{{12}}}} \\ &{}+O \bigl(\bigl( \vert X \vert + \vert Y \vert \bigr)^{4} \bigr) \end{aligned}$$

and

$$\begin{aligned} \breve{G}(X,Y)={}& \biggl( { \frac{ ( \ell -v_{{11}} ) v_{{16}}}{v_{{12}}\wp }}-{ \frac{v_{{26}}}{\wp }} \biggr) {P}^{3}+ \biggl( { \frac{ ( \ell -v_{{11} } ) v_{{17}}}{v_{{12}}\wp }}-{\frac{v_{{27}}}{\wp }} \biggr) {P}^{2}Z \\ &{}+ \biggl( { \frac{ ( \ell -v_{{11}} ) v_{{13}}}{v_{{12}}\wp }}-{ \frac{v_{{23}}}{\wp }} \biggr) {P}^{2} + \biggl( { \frac{ ( \ell -v_{{11}} ) v_{{18}}}{v_{{12}}\wp }}-{ \frac{v_{{28}}}{\wp }} \biggr) P{Z}^{2} \\ &{}+ \biggl( { \frac{ ( \ell -v_{{11}} ) v_{{14}}}{v_{{12}}\wp }}-{ \frac{v_{{24}}}{\wp }} \biggr) PZ + \biggl( { \frac{ ( \ell -v_{{11}} ) v_{{19}}}{v_{{12}}\wp }}-{ \frac{v_{{29}}}{\wp }} \biggr) {Z}^{3} \\ &{}+ \biggl( { \frac{ ( \ell -v_{{11}} ) v_{{15}}}{v_{{12}}\wp }}-{ \frac{v_{{25}}}{\wp }} \biggr) {Z}^{2} +O \bigl(\bigl( \vert X \vert + \vert Y \vert \bigr)^{4} \bigr) \end{aligned}$$

with $P=v_{12}X$ and $Z=(\ell -v_{11})X-\wp Y$. Hence by using the standard theory of normal form for analysis of bifurcation we can calculate the first Lyapunov exponent at $(X,Y)=(0,0)$ as follows:

$$\begin{aligned} \Omega = \biggl( \biggl[-\operatorname{Re} \biggl( \frac{(1-2\xi _{1})\xi _{2}^{2}}{1-\xi _{1}}\theta _{20} \theta _{11} \biggr)-\frac{1}{2} \vert \theta _{11} \vert ^{2}- \vert \theta _{02} \vert ^{2}+\operatorname{Re}(\xi _{2} \theta _{21}) \biggr] \biggr)_{\breve{h}=0}, \end{aligned}$$

where

$$\begin{aligned} &\theta _{20}=\frac{1}{8} \bigl[\breve{F}_{XX}- \breve{F}_{YY}+2 \breve{G}_{XY}+ i (\breve{G}_{XX}- \breve{G}_{YY}-2\breve{F}_{XY} ) \bigr], \\ &\theta _{11}=\frac{1}{4} \bigl[\breve{F}_{XX}+ \breve{F}_{YY}+ i ( \breve{G}_{XX}+\breve{G}_{YY} ) \bigr], \\ &\theta _{02}=\frac{1}{8} \bigl[\breve{F}_{XX}- \breve{F}_{YY}-2 \breve{G}_{XY}+ i (\breve{G}_{XX}- \breve{G}_{YY}+2\breve{F}_{XY} ) \bigr], \\ &\theta _{21}=\frac{1}{16} (\breve{F}_{XXX}+ \breve{F}_{XYY}+ \breve{G}_{XXY}+\breve{G}_{YYY} ) \\ &\phantom{\theta _{21}=}{}+\frac{i}{16} (\breve{G}_{XXX}+\breve{G}_{XYY}- \breve{F}_{XXY}- \breve{F}_{YYY} ). \end{aligned}$$

Due to aforementioned analysis, we have the following theorem (see [45–50]).

### Theorem 4.1

*Assume that* (3.7), (3.8), *and* (3.9) *are satisfied and* $\Omega \neq 0$. *Then the unique positive fixed point* $(\underline{p},\underline{z})$ *of system* (1.5) *undergoes Neimark–Sacker bifurcation*. *Additionally*, *if* $\Omega <0$, *then for* $h>\breve{h}$, *an attracting invariant closed curve bifurcates from the fixed point* $(\underline{p},\underline{z})$, *and if* $\Omega >0$, *then for* $h<\breve{h,}$ *a repelling invariant closed curve bifurcates from the fixed point* $(\underline{p},\underline{z})$.

## Modified hybrid control strategy for controlling bifurcation and chaos

Generally, discrete-time systems are more complex to analyze as compared to a continuous-time one. For survival of life in any environment, it is necessary that the population does not experience any irregular situation. Hence, for controlling accidental uneven and unstable behavior in any mathematical system, chaos control is considered to be an applied tool for evading this complex and chaotic behavior [51–53]. In this part of the paper, we study a feedback control method with parameter perturbation to move unstable and irregular trajectories toward the stable trajectories. The most useful and well-known method in the field of chaos is given by Ott et al. [51] to control period-doubling bifurcation, which is known as OGY method. Later on, numerous strategic control methods are developed (see [53]). Here we consider a modified hybrid control method to control the Neimark–Sacker bifurcation and chaos. Furthermore, this mathematical method is well applicable to every discrete-time system experiencing the period-doubling bifurcation and chaos. Originally, a hybrid method was proposed by Liu et al. [52]. Moreover, it was developed to control the period-doubling bifurcation (see [54, 55]). Here we reformed the existing hybrid control technique [52] to control the Neimark–Sacker bifurcation and chaos. Furthermore, the newly developed technique has shown better results for almost every discrete dynamical system. Consider the following *n*-dimensional discrete dynamical system:

5.1 $$\begin{aligned} Z_{n+1}=g(Z_{n},\mu ) \end{aligned}$$

with $Z_{n} \in \Re ^{n}$, $n \in Z$, and the parameter $\mu \in \Re $ for which system (5.1) experiences the bifurcation. The purpose of proposing the reformed method for controlling the bifurcation is recapturing the extreme range of stable region in (5.1) by lessening the length of unstable region. Hence we present the following generalized hybrid control method by applying state feedback along with parameter perturbation;

5.2 $$\begin{aligned} Z_{n+k}=L^{3} g^{(\hbar )}(Z_{n}, \mu )+ \bigl(1-L^{3}\bigr)Z_{n}, \end{aligned}$$

where $\hbar \in Z$, and $0<{L}<1$ is a parameter for controlling the bifurcation appearing in (5.2). In addition, $g^{(\hbar )}$ is the *k*th value of $g(\cdot )$. By application of (5.2) to system (1.5) we get the following system:

5.3 $$\begin{aligned} \textstyle\begin{cases} p_{n+1}=L^{3}( \frac{(1+hr)p_{n}}{1+h(\frac{r}{k}p_{n}+\frac{\alpha z_{n}}{a+p_{n}}+m_{1}p^{2}_{n}+q_{1}E)})+(1-L^{3})p_{n}, \\ z_{n+1}=L^{3}( \frac{(1+h\frac{\beta p_{n}}{a+p_{n}})z_{n}}{1+h(\frac{\rho p_{n}}{a+p_{n}}+\delta +m_{2}z_{n}+q_{2}E)})+(1-L^{3})z_{n}. \end{cases}\displaystyle \end{aligned}$$

Furthermore, systems (5.3) and (1.5) have the same constant solutions. Additionally, the Jacobian matrix of (5.3) about $(\underline{p},\underline{z})$ is given as follows:

5.4 $$\begin{aligned} \begin{pmatrix} 1- \frac{h L^{3} \underline{p} ((a-k) r+e k q_{1}+\underline{p} (2 r+k m_{1} (2 a+3 \underline{p} ) ) )}{k (1+h r) (a+\underline{p} )} & - \frac{h L^{3} \alpha \underline{p}}{(1+h r) (a+\underline{p} )} \\ \frac{h L^{3} \underline{z} (-\beta +\delta +\rho +e q_{2}+m_{2} \underline{z} )^{2}}{a (\beta +h \beta \delta -\rho +e h \beta q_{2}+h \beta m_{2} \underline{z} )} & \frac{\beta +h \beta \delta -\rho +e h \beta q_{2}+h (\beta -L^{3} \beta +L^{3} \rho ) m_{2} \underline{z}}{\beta +h \beta \delta -\rho +e h \beta q_{2}+h \beta m_{2} \underline{z}} \end{pmatrix}. \end{aligned}$$

The following theorem describes a necessary and sufficient condition for local stability of system (5.3) about $(\underline{p},\underline{z})$.

### Theorem 5.1

*The positive constant solution* $(\underline{p},\underline{z})$ *of system* (5.3) *is locally asymptotically stable if and only if*

$$\begin{aligned} \mid \operatorname{Tr} \mid < 1+Dt< 2, \end{aligned}$$

*where* Tr *and* *Dt* *are the trace and determinant of* (5.4), *respectively*.

For the understanding of limitation of modified hybrid control technique, we have the following remark.

### Remark

Like the hybrid method [52], the modified hybrid method (5.2) is feasible and efficient for those discrete-time mathematical models for which the stepsize parameter is taken as a bifurcation parameter.

## Hybrid control of Neimark–Sacker bifurcation

In this section, we apply the hybrid technique [52] to system (1.5) to control the Neimark–Sacker bifurcation. Moreover, this method is used as control strategy by many researchers for controlling the period-doubling bifurcation, Neimark–Sacker bifurcation, and chaos under the effects of period-doubling bifurcation (see [54, 55]). By application of a hybrid method [52] to system (1.5) we get the following system:

6.1 $$\begin{aligned} \textstyle\begin{cases} p_{n+1}=S_{1}( \frac{(1+hr)p_{n}}{1+h(\frac{r}{k}p_{n}+\frac{\alpha z_{n}}{a+p_{n}}+m_{1}p^{2}_{n}+q_{1}E)})+(1-S_{1})p_{n}, \\ z_{n+1}=S_{1}( \frac{(1+h\frac{\beta p_{n}}{a+p_{n}})z_{n}}{1+h(\frac{\rho p_{n}}{a+p_{n}}+\delta +m_{2}z_{n}+q_{2}E)})+(1-S_{1})z_{n}, \end{cases}\displaystyle \end{aligned}$$

where $0< S_{1}<1$ is a control parameter. Furthermore, systems (6.1) and (1.5) have the same constant solutions. Additionally, the Jacobian matrix of (6.1) about $(\underline{p},\underline{z})$ is

6.2 $$\begin{aligned} \begin{pmatrix} 1- \frac{h S_{1} \underline{p} ((a-k) r+e k q_{1}+\underline{p} (2 r+k m_{1} (2 a+3 \underline{p} ) ) )}{k (1+h r) (a+\underline{p} )} & - \frac{h S_{1} \alpha \underline{p}}{(1+h r) (a+\underline{p} )} \\ \frac{h S_{1} \underline{z} (-\beta +\delta +\rho +e q_{2}+m_{2} \underline{z} )^{2}}{a (\beta +h \beta \delta -\rho +e h \beta q_{2}+h \beta m_{2} \underline{z} )} & \frac{\beta +h \beta \delta -\rho +e h \beta q_{2}+h (\beta -S_{1} \beta +S_{1} \rho ) m_{2} \underline{z}}{\beta +h \beta \delta -\rho +e h \beta q_{2}+h \beta m_{2} \underline{z}} \end{pmatrix}. \end{aligned}$$

## Numerical simulation

In this section, we numerically study the dynamics of (1.5). This study is a direct verification of our theoretical analysis and analytic results we have proved in the previous sections. Particularly, in this section, we study the existence and direction of Neimark–Sacker bifurcation by using numeric values of the parameters. In addition, in this section, we take the initial conditions in the least neighborhood of the equilibrium point $(\underline{p},\underline{z})$ for each case study.

### Example 7.1

Let $a=2.0099, q_{1}=0.0189, q_{2}=1.2994, r=10.5923, E=0.9959, k=1.3997, \beta =98.499, \alpha =2.9999, \delta =0.0384, \rho =10.5842, m_{1}=0.6222, m_{2}=0.4422, p_{0}=0.105348, z_{0}=6.8884251$, and $h\in (0,1)$]. In this case the extinction equilibrium and nonextinction equilibrium for zooplankton population are $(\underline{x},0)=(1.26553,0)$ and $(\underline{p},\underline{z})=(0.1053484, 6.888425)$, respectively. Then from system (1.5) we have

$$\begin{aligned} \limsup_{n \to \infty }{p_{n}}\leq k=1.3997. \end{aligned}$$

Then by using the value $k=1.3997$ in the second equation of system (1.5) we get

$$\begin{aligned} \limsup_{n \to \infty }{z_{n}}\leq \frac{k(\beta -\rho )}{m_{2}(k+a)}=81.61590194902372. \end{aligned}$$

Hence we have $(0.1053484, 6.888425) \in [0,k ]\times [0, \frac{k(\beta -\rho )}{m_{2}(k+a)} ]$ for $\beta >\rho $, which verifies Theorem 2.1.

Additionally, we have

$$\begin{aligned} f(0)=\frac{a(r-q_{1}E)}{\alpha }=7.084113606170539>0 \end{aligned}$$

and

$$\begin{aligned} F(0)= \frac{a(\delta +m_{2}f(0)+q_{2}E)}{(\beta -\rho )-(\delta +m_{2}f(0)+q_{2}E)}=0.10754185654404144>0. \end{aligned}$$

Furthermore, for $\lambda =1.3997$, we have $(\beta -\rho )=88.34599$ and $(\delta +m_{2}f(\lambda )+q_{2}E)=4.465067496648613$. Then $(\beta -\rho )>(\delta +m_{2}f(\lambda )+q_{2}E)$, and we get

$$\begin{aligned} F(\lambda )= \frac{a(\delta +m_{2}f(\lambda )+q_{2}E)}{(\beta -\rho )-(\delta +m_{2}f(\lambda )+q_{2}E)}- \lambda =-1.2921581434559586< 0, \end{aligned}$$

where

$$\begin{aligned} f(\lambda )=-\frac{(a+\lambda )(m_{1}r^{2}+q_{1}E)}{\alpha }=-79.36413532682512< 0. \end{aligned}$$

Moreover, we have

$$\begin{aligned} F'(\lambda )=-1+ \frac{a(\beta -\rho )(m_{2}f'(\lambda ))}{ ((\beta -\rho )-(\delta +m_{2}f(\lambda )+q_{2}E) )^{2}}=-1.0580185510185083< 0, \end{aligned}$$

where

$$\begin{aligned} f'(\lambda )=-\frac{(a+\lambda )(\frac{r}{k}+2m_{1}\lambda )}{\alpha }+ \frac{r-\frac{r\lambda }{k}-m_{1}\lambda ^{2}-q_{1}E}{\alpha }=-10.993342080620321< 0, \end{aligned}$$

which verifies Theorem 2.2.

### Example 7.2

Let $a=2.0099, q_{1}=0.0189, q_{2}=1.2994, r=10.5923, E=0.9959, k=1.3997, \beta =98.499, \alpha =2.9999, \delta =0.0384, \rho =10.5842, m_{1}=0.6222, m_{2}=0.4422, p_{0}=0.105348, z_{0}=6.8884251$, and $h\in (0,1)$]. Then system (1.5) takes the form

7.1 $$\begin{aligned} \textstyle\begin{cases} p_{n+1}= \frac{(1+10.5923 h)p_{n}}{1+h(\frac{10.5923}{1.3997}p_{n}+\frac{2.9999 z_{n}}{2.0099+p_{n}}+0.6222p^{2}_{n}+0.0188)}, \\ z_{n+1}= \frac{(1+h\frac{98.499 p_{n}}{2.0099+p_{n}})z_{n}}{1+h(\frac{10.5842 p_{n}}{2.0099+p_{n}}+0.0384+0.4422z_{n}+1.2941)}. \end{cases}\displaystyle \end{aligned}$$

Additionally, in this case the extinction equilibrium and nonextinction equilibrium for zooplankton population are $(\underline{x},0)=(1.26553,0)$ and $(\underline{p},\underline{z})=(0.1053484, 6.888425)$, respectively. In this case the graphical behavior of both population variables is shown in Fig. 2. In addition, Fig. 2(c) represents the maximum Lyapunov exponent for system (7.1). In Fig. 3, some phase portraits are given, where *h* varies in $]0,1[$. We can easily see that there exists the Neimark–Sacker bifurcation when *h* certainly passes through $h=0.38022$ (see Fig. 3(b)). For the aforementioned values of parameters, the Jacobian matrix $V_{2}(\underline{p},\underline{z})$ for system (7.1) is

$$\begin{aligned} &V_{2}(0.1053484433,6.88842511)\\ &\quad= \begin{pmatrix} 0.9751639697174742 & -0.01143564868241927 \\ 36.869495419357726 & 0.5871690414953413 \end{pmatrix}. \end{aligned}$$

Moreover, the characteristic equation $\mathbb{M}(\xi )=0$ for $V_{2}(0.1053484433,6.88842511)$ is

7.2 $$\begin{aligned} \xi ^{2}-1.8038899298174336\xi +1=0. \end{aligned}$$

Solving (7.2), we get $\xi _{1}=0.7811665056064+0.6196705420078 i$ and $\xi _{2}= 0.7811665056064-0.6196705420078i$ with $|\xi _{1}|=|\xi _{1}|=1$. In addition, we have

$$\begin{aligned} \mathbb{M}(-1)=3.556545701326458>0 \end{aligned}$$

and

$$\begin{aligned} \mathbb{M}(1)=0.43187967890082724>0. \end{aligned}$$

Now from (4.3) we have

$$\begin{aligned} \breve{f}(P,Z)={}& 0.105348+ 0.974755 P- 0.0116237 Z- 0.256573 {P}^{2 }- 0.099269 PZ \\ &{}+ 0.001282 {Z}^{2}- 0.142822 {P}^{3}+ 0.10288 {P}^{2}Z+ 0.010039 P{Z}^{2} \\ &{}- 0.000141506 {Z}^{3}+O \bigl(\bigl( \vert P \vert + \vert Z \vert \bigr)^{4}\bigr) \end{aligned}$$

and

$$\begin{aligned} \breve{g}(P,Z)={}& 6.88842+ 0.574993 Z+ 37.95688 P- 43.12450 {P}^{2}+ 3.450296 PZ \\ &{}-0.0354763 {Z}^{2}+ 48.99565 {P}^{3}- 2.366456 {P}^{2}Z- 0.1893441 P{Z}^{2} \\ &{}+ 0.00218884 {Z}^{3}+O \bigl(\bigl( \vert P \vert + \vert Z \vert \bigr)^{4}\bigr). \end{aligned}$$

Finally, when system (4.3) is converted into the canonical form (4.6), we obtain the matrix

$$\begin{aligned} \begin{bmatrix} \frac{1}{v_{12}} & 0 \\ \frac{\ell -v_{11}}{\wp v_{12} } & -\frac{1}{\wp } \end{bmatrix}= \begin{bmatrix} - 0.01143564869&0 \\ - 0.1939974644&- 0.6196705420078615\end{bmatrix} \end{aligned}$$

with

$$\begin{aligned} \begin{bmatrix} \frac{1}{v_{12}} & 0 \\ \frac{\ell -v_{11}}{\wp v_{12} } & -\frac{1}{\wp } \end{bmatrix}^{-1}= \begin{bmatrix} - 87.4458482512197577& 0.0 \\ 27.3762776879404903&- 1.61376075222132043 \end{bmatrix}. \end{aligned}$$

Furthermore, from (4.8) we have

$$\begin{aligned} \breve{F}(X,Y)={}&22.073323320819 {P}^{2}+ 8.5402951060818 PZ- 0.1103358266003 {Z}^{2} \\ &{}+12.2871550765 {P}^{3}-8.85102538857 {P}^{2}Z- 0.8636988319698 P{Z}^{2} \\ &{}+ 0.012173994624 {Z}^{3}+O \bigl(\bigl( \vert X \vert + \vert Y \vert \bigr)^{4}\bigr) \end{aligned}$$

and

$$\begin{aligned} \breve{G}(X,Y)={}&61.114564266701 {P}^{2}- 8.141785570023 PZ+0.0908219975961 {Z}^{2} \\ &{}- 81.22561954155 {P}^{3}+6.52880078485 {P}^{2}Z+ 0.571452207625 P{Z}^{2} \\ &{}- 0.0072969596695 {Z}^{3}+O \bigl(\bigl( \vert X \vert + \vert Y \vert \bigr)^{4}\bigr). \end{aligned}$$

**Figure 1:**
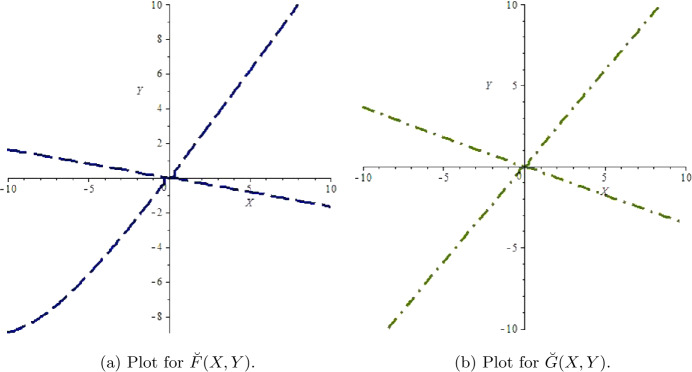
Plots for $\breve{F}(X,Y)$ and $\breve{G}(X,Y)$ for $a=2.0099$, $q_{1}=0.0189$, $q_{2}=1.2994$, $r=10.5923$, $E=0.9959$, $k=1.3997$, $\beta =98.499$, $\alpha =2.9999$, $\delta =0.0384$, $\rho =10.5842$, $m_{1}=0.6222$, $m_{2}=0.4422$, and $h\in (0,1)$

Additionally, plots for $\breve{F}(X,Y)$ and $\breve{G}(X,Y)$ with solution at $(0,0)$ are presented in Figs. 1(a) and 1(b), respectively, where $P=(-0.1162370758)X$ and $Z=(-0.1998810610)X- (0.6334408575)Y$. Finally, we get

$$\begin{aligned} &\theta _{20}=\frac{1}{8} \bigl[\breve{F}_{XX}- \breve{F}_{YY}+2 \breve{G}_{XY}+ i (\breve{G}_{XX}- \breve{G}_{YY}-2\breve{F}_{XY} ) \bigr]=0.005831 -0.019066 i, \\ &\theta _{11}=\frac{1}{4} \bigl[\breve{F}_{XX}+ \breve{F}_{YY}+ i ( \breve{G}_{XX}+\breve{G}_{YY} ) \bigr]=-0.01195+0.013959 i, \\ &\theta _{02}=\frac{1}{8} \bigl[\breve{F}_{XX}- \breve{F}_{YY}-2 \breve{G}_{XY}+ i (\breve{G}_{XX}- \breve{G}_{YY}+2\breve{F}_{XY} ) \bigr]=0.02389 -0.00182 i, \\ &\theta _{21}=\frac{1}{16} (\breve{F}_{XXX}+ \breve{F}_{XYY}+ \breve{G}_{XXY}+\breve{G}_{YYY} ) \\ &\phantom{\theta _{21}=}{}+\frac{i}{16} (\breve{G}_{XXX}+\breve{G}_{XYY}- \breve{F}_{XXY}- \breve{F}_{YYY} )=0.00755 +0.00574 i, \end{aligned}$$

and

$$\begin{aligned} \Omega ={}& \biggl( \biggl[-\operatorname{Re} \biggl( \frac{(1-2\xi _{1})\xi _{2}^{2}}{1-\xi _{1}}\theta _{20} \theta _{11} \biggr)-\frac{1}{2} \vert \theta _{11} \vert ^{2}- \vert \theta _{02} \vert ^{2}+\operatorname{Re}(\xi _{2} \theta _{21}) \biggr] \biggr)_{\hat{h}=0} \\ ={}&{-}0.00138464412< 0. \end{aligned}$$

**Figure 2:**
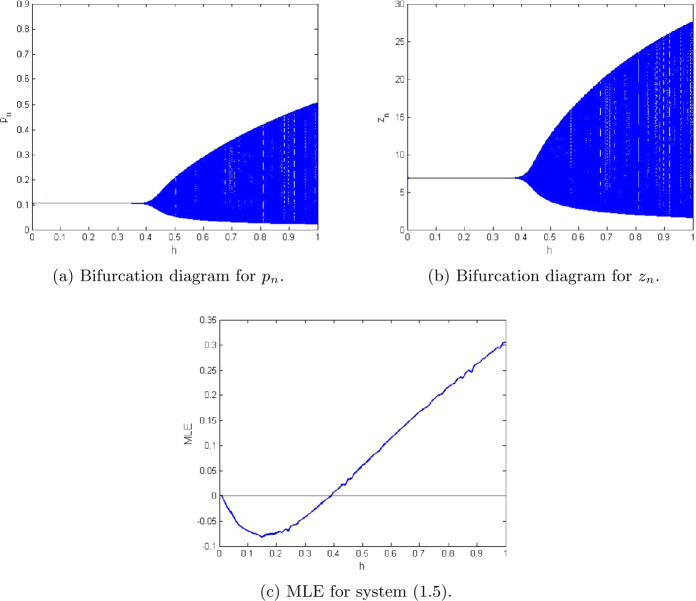
Plots of system (1.5) for $a=2.0099$, $q_{1}=0.0189$, $q_{2}=1.2994$, $r=10.5923$, $E=0.9959$, $k=1.3997$, $\beta =98.499$, $\alpha =2.9999$, $\delta =0.0384$, $\rho =10.5842$, $m_{1}=0.6222$, $m_{2}=0.4422$, and $h\in (0,1)$

**Figure 3:**
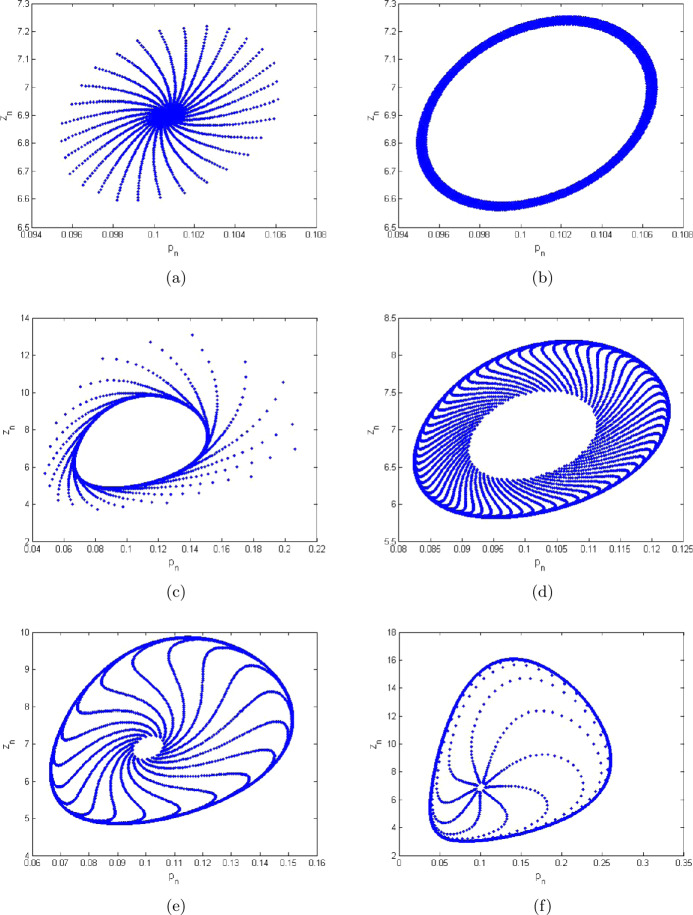
Phase portraits of system (1.5) for $a=2.0099$, $q_{1}=0.0189$, $q_{2}=1.2994$, $r=10.5923$, $E=0.9959$, $k=1.3997$, $\beta =98.499$, $\alpha =2.9999$, $\delta =0.0384$, $\rho =10.5842$, $m_{1}=0.6222$, $m_{2}=0.4422$, and $h\in (0,1)$

Hence the condition for the existence of Neimark–Sacker bifurcation is satisfied (see Theorem 4.1).

### Example 7.3

**Figure 4:**
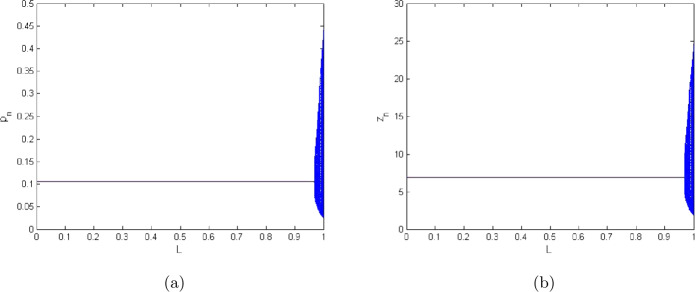
Controlled diagrams for system (7.1) for $a=2.0099$, $q_{1}=0.0189$, $q_{2}=1.2994$, $r=10.5923$, $E=0.9959$, $k=1.3997$, $\beta =98.499$, $\alpha =2.9999$, $\delta =0.0384$, $\rho =10.5842$, $m_{1}=0.6222$, $m_{2}=0.4422$, $h=0.699909$ and $L\in (0,1)$

This example is related to the study of control of Neimark–Sacker bifurcation by using generalized hybrid technique (5.2). To show the effectiveness of generalized technique, we have used the same values of parameters as in Example 7.1. Consider the following system of difference equations:

7.3 $$\begin{aligned} \textstyle\begin{cases} p_{n+1}=L^{3}( \frac{(1+10.5923 h)p_{n}}{1+h(\frac{10.5923}{1.3997}p_{n}+\frac{2.9999 z_{n}}{2.0099+p_{n}}+0.6222p^{2}_{n}+0.0188)})+(1-L^{3})p_{n}, \\ z_{n+1}=L^{3}( \frac{(1+h\frac{98.499 p_{n}}{2.0099+p_{n}})z_{n}}{1+h(\frac{10.5842 p_{n}}{2.0099+p_{n}}+0.0384+0.4422z_{n}+1.2941)})+(1-L^{3})z_{n}, \end{cases}\displaystyle \end{aligned}$$

where $a=2.0099, q_{1}=0.0189, q_{2}=1.2994, r=10.5923, E=0.9959, k=1.3997, \beta =98.499, \alpha =2.9999, \delta =0.0384, \rho =10.5842, m_{1}=0.6222, m_{2}=0.4422, h=0.699909$. In addition, $0< L<1$ is the control parameter. Furthermore, for system (7.3), we have $(\underline{p},\underline{z})=(0.1053484433, 6.88842511)$, which is a unique positive constant solution of the original system (1.5). Additionally, controlled diagrams for zooplankton and phytoplankton populations by using models (7.3) are shown in Figs. 4(b) and 4(a), respectively. Finally, we can see that the stability of initial system (1.5) is victoriously regained for large range of control parameter by using the generalized hybrid control method (see Fig. 4).

### Example 7.4

This example is related to the study of control of Neimark–Sacker bifurcation by using a hybrid technique [52] of control. Consider the system of difference equations

**Figure 5:**
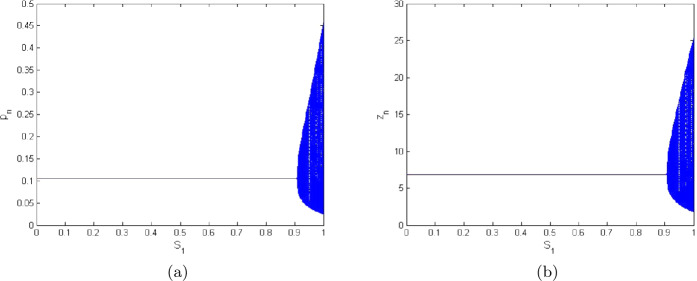
Controlled diagrams for system (7.4) for $a=2.0099$, $q_{1}=0.0189$, $q_{2}=1.2994$, $r=10.5923$, $E=0.9959$, $k=1.3997$, $\beta =98.499$, $\alpha =2.9999$, $\delta =0.0384$, $\rho =10.5842$, $m_{1}=0.6222$, $m_{2}=0.4422$, $h=0.699909$, and $S_{1}\in (0,1)$

7.4 $$\begin{aligned} \textstyle\begin{cases} p_{n+1}=S_{1}( \frac{(1+10.5923 h)p_{n}}{1+h(\frac{10.5923}{1.3997}p_{n}+\frac{2.9999 z_{n}}{2.0099+p_{n}}+0.6222p^{2}_{n}+0.0188)})+(1-S_{1})p_{n}, \\ z_{n+1}=S_{1}( \frac{(1+h\frac{98.499 p_{n}}{2.0099+p_{n}})z_{n}}{1+h(\frac{10.5842 p_{n}}{2.0099+p_{n}}+0.0384+0.4422z_{n}+1.2941)})+(1-S_{1})z_{n}, \end{cases}\displaystyle \end{aligned}$$

where $a=2.0099, q_{1}=0.0189, q_{2}=1.2994, r=10.5923, E=0.9959, k=1.3997, \beta =98.499, \alpha =2.9999, \delta =0.0384, \rho =10.5842, m_{1}=0.6222, m_{2}=0.4422, h=0.699909$. In addition, $0< S_{1}<1$ is the control parameter. Furthermore, for system (7.4), we have $(\underline{p},\underline{z})=(0.1053484433, 6.88842511)$, which is a unique positive constant solution of the original system (1.5). Additionally, controlled diagrams for zooplankton and phytoplankton populations for system (7.4) are respectively shown in Figs. 5(b) and 5(a).

### Example 7.5

In this example, we compare the generalized hybrid method and hybrid method [52]. From Examples 7.3 and 7.4 we consider two discrete-time models (7.3) and (7.4), respectively. Moreover, in this case, we have taken $L, S_{1}\in ]0,1[$ and $a=2.0099, q_{1}=0.0189, q_{2}=1.2994, r=10.5923, E=0.9959, k=1.3997, \beta =98.499, \alpha =2.9999, \delta =0.0384, \rho =10.5842, m_{1}=0.6222, m_{2}=0.4422$, and $h\in (0,1)$.

Form both systems (7.3) and (7.4) we get $(\underline{p},\underline{z})=(0.1053484433, 6.88842511)$ as a nique positive fixed point. Additionally, from Table 1 we can observe that $|I_{1}|>|I_{2}|$ for each variation of the parameter $h\in ]0,1[$, where $I_{1}$ and $I_{2}$ are the controlled intervals corresponding to the controlled systems (7.3) and (7.4), respectively. Hence we can see from Table 1 that the generalized hybrid method (5.2) is much better than the old hybrid method [52].

**Table 1:** Comparison of the modified hybrid method (5.2) and hybrid method [52] for $L, S_{1}\in ]0,1[$ and $a=2.0099$, $q_{1}=0.0189$, $q_{2}=1.2994$, $r=10.5923$, $E=0.9959$, $k=1.3997$, $\beta =98.499$, $\alpha =2.9999$, $\delta =0.0384$, $\rho =10.5842$, $m_{1}=0.6222$, $m_{2}=0.4422$, $h\in (0,1)$

*h*∈]0,1[	Controlled interval $I_{1}$ for (7.3)	Controlled interval $I_{2}$ for (7.4)
0.48889569	0<*L*<0.99288325491279	$0< S_{1}<0.97880134847096$
0.58889569	0<*L*<0.983282763587931	$0< S_{1}<0.95068201684448$
0.68889569	0<*L*<0.97635403882826	$0< S_{1}<0.93072628972275$
0.78889569	0<*L*<0.97111704728074	$0< S_{1}<0.91582972183557$
0.88889569	0<*L*<0.96701917628990	$0< S_{1}<0.90428485868004$
0.98889569	0<*L*<0.96372500134675	$0< S_{1}<0.89507489723917$

### Example 7.6

**Figure 6:**
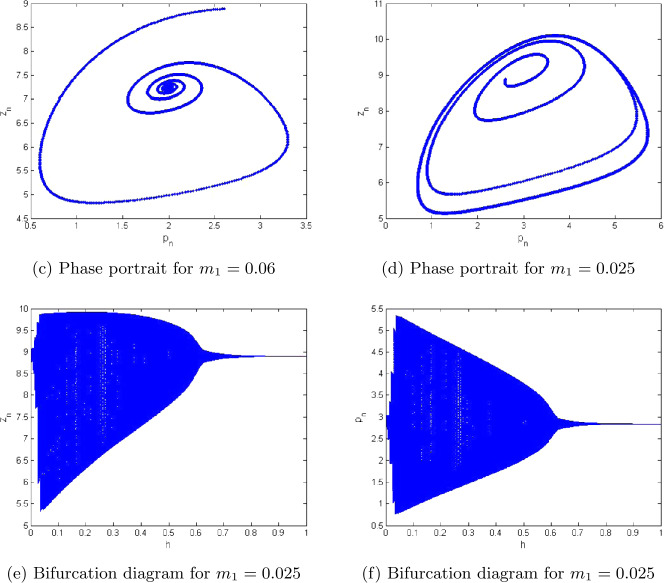
Bifurcation diagrams and phase portraits for system (1.5) for $a=2.1$, $q_{1}=0.09$, $q_{2}=0.3$, $r=1.5$, $c=0.14$, $k=100$, $\beta =0.5$, $\alpha =0.69$, $\delta =0.001$, $\rho =0.1$, $m_{2}=0.021$, and $h=0.0399909$

**Figure 7:**
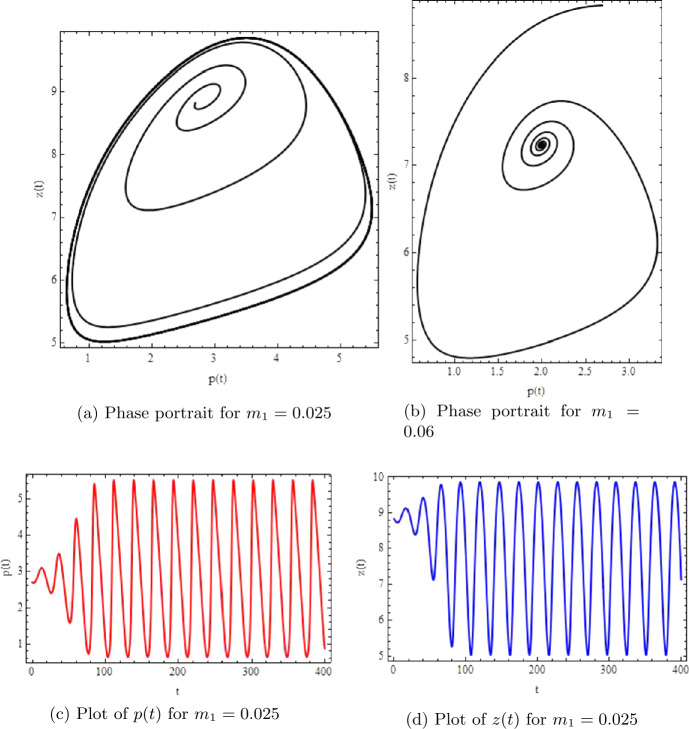
Plots and phase portraits for system (1.3) for $a=2.1$, $q_{1}=0.09$, $q_{2}=0.3$, $r=1.5$, $c=0.14$, $k=100$, $\beta =0.5$, $\alpha =0.69$, $\delta =0.001$, $\rho =0.1$, and $m_{2}=0.021$

In this example, we compare the dynamics of systems (1.3) and (1.5). For case (i), we take $a=2.1, q_{1}=0.09, q_{2}=0.3, r=1.5, c=0.14, k=100, \beta =0.5, \alpha =0.69, \delta =0.001, \rho =0.1, m_{2}=0.021$, and $m_{1}=0.06$. Then we get the fixed point $(\underline{p},\underline{z})=(2.3059, 7.38854)$, which is a unique positive constant solution of (1.3) and (1.5). Moreover, for the initial conditions $p_{0}=2.3059$ and $z_{0}=7.38854$, Figs. 6(a) and 7(b) are plotted for systems (1.5) and (1.3), respectively. Consequently, we can see that systems (1.5) and (1.3) are stable at $(\underline{p},\underline{z})=(2.3059, 7.38854)$ for $m_{1}=0.06$ (see Figs. 6(a) and 7(b)). In addition, for case (ii), we take $m_{1}=0.025$, $a=2.1, q_{1}=0.09, q_{2}=0.3, r=1.5, c=0.14, k=100, \beta =0.5, \alpha =0.69, \delta =0.001, \rho =0.1, m_{2}=0.021$. We get the fixed point $(\underline{p},\underline{z})=(2.8059, 8.8854)$, which is a unique positive constant solution of (1.3) and (1.5). Hence we can see that both systems (1.3) and (1.5) are unstable at $(\underline{p},\underline{z})=(2.8059, 8.8854)$ (see Figs. 6(b) and 7(a)). Finally, Fig. 6(c), (d) shows the existence of Neimark–Sacker bifurcation in system (1.5) for lower values of stepsize *h*, and Fig. 7(c), (d) shows that both variables $p(t)$ and $z(t)$ from system (1.3) are unstable at $(\underline{p},\underline{z})=(2.8059, 8.8854)$ (see [25]).

## Concluding remarks

We study the dynamics of a discrete-time phytoplankton–zooplankton model [25]. Firstly, by implementing nonstandard difference scheme we obtained a discrete-time version of the model presented in [25]. We prove that each solution of system (1.5) is bounded and contained in a rectangular region (Theorem 2.1). Moreover, we show that there exist a unique positive fixed point of system (1.5), which is contained in that rectangular region (Theorem 2.2). In addition, we discuss the local stability of system (1.5) about each its fixed point. To prove the complexity in system (1.5), we show the existence of Neimark–Sacker bifurcation for the unique positive fixed point. Neimark–Sacker bifurcation is effectively controlled by using two different methods, the generalized hybrid control method and hybrid control method [52]. To verify our theoretical investigations, we provide a comprehensive numerical simulation at the end of the paper. In Example 7.1, we provide a numeric validation of Theorems 2.1 and 2.2. We numerically and graphically show that system (1.5) experiences a Neimark–Sacker bifurcation for large range of stepsize *h*. In addition, we provide two examples related to the control of Neimark–Sacker bifurcation. For explanation of our theoretical results and comparison of the dynamics of obtained discrete-time model (1.5) with its continuous counterpart (1.3), we provide some motivating numerical examples. Moreover, from numerical study in Example 7.6 we can see that the obtained system (1.5) and its continuous-time counterpart (1.3) are stable and unstable for the same parameter values. Hence the dynamical consistency of our obtained system (1.5) can be seen from numerical study. For the study of both cases in Example 7.6, the parametric values are taken from [25]. We show that systems (1.3) and (1.5) are stable for the values of $m_{1}$ greater than or equal to $m_{1}=0.06$ and unstable for $m_{1}<0.06$. In addition, our numerical study showed that the generalized hybrid method (5.2) is better than the hybrid method [52]. In addition, it is based on feedback control and it has brought back the stability of system (1.5) for large ranges of parameters. Moreover, from the numerical study we can see that the generalized hybrid method (5.2) is suitable for controlling the Neimark–Sacker bifurcation. At last, in Table 1, we provide a comparison of the generalized hybrid method (5.2) and hybrid method [52]. Moreover, from Fig. 4, Fig. 5, and Table 1 we can see that the generalized hybrid control technique restores the stability of system (1.5) for the maximal range of the control parameter.

## Data Availability

Not applicable.
